# Smarca4 deficiency induces Pttg1 oncogene upregulation and hyperproliferation of tubular and interstitial cells during kidney development

**DOI:** 10.3389/fcell.2023.1233317

**Published:** 2023-09-04

**Authors:** Jinshu Xu, Xianxiao Zhou, Ting Zhang, Bin Zhang, Pin-Xian Xu

**Affiliations:** ^1^ Department of Genetics and Genomic Sciences, Icahn School of Medicine at Mount Sinai, New York, NY, United States; ^2^ Mount Sinai Center for Transformative Disease Modeling, Icahn School of Medicine at Mount Sinai, New York, NY, United States; ^3^ Department of Pharmacological Sciences, Icahn School of Medicine at Mount Sinai, New York, NY, United States; ^4^ Department of Cell, Developmental and Regenerative Biology, Icahn School of Medicine at Mount Sinai, New York, NY, United States

**Keywords:** SWI/SNF chromatin remodeling complex, Smarca4/Brg1, *Wnt4*, nephron tubulogenesis, renal interstitium, *Pttg1*, fibrosis

## Abstract

Kidney formation and nephrogenesis are controlled by precise spatiotemporal gene expression programs, which are coordinately regulated by cell-cycle, cell type-specific transcription factors and epigenetic/chromatin regulators. However, the roles of epigenetic/chromatin regulators in kidney development and disease remain poorly understood. In this study, we investigated the impact of deleting the chromatin remodeling factor *Smarca4 (Brg1)*, a human Wilms tumor-associated gene, in *Wnt4*-expressing cells. *Smarca4* deficiency led to severe tubular defects and a shortened medulla. Through unbiased single-cell RNA sequencing analyses, we identified multiple types of *Wnt4* ^*Cre*^-labeled interstitial cells, along with nephron-related cells. *Smarca4* deficiency increased interstitial cells but markedly reduced tubular cells, resulting in cells with mixed identity and elevated expression of cell-cycle regulators and genes associated with extracellular matrix and epithelial-to-mesenchymal transition/fibrosis. We found that *Smarca4* loss induced a significant upregulation of the oncogene *Pttg1* and hyperproliferation of *Wnt4* ^*Cre*^-labeled cells. These changes in the cellular state could hinder the cellular transition into characteristic tubular structures, eventually leading to fibrosis. In conclusion, our findings shed light on novel cell types and genes associated with *Wnt4* ^*Cre*^-labeled cells and highlight the critical role of Smarca4 in regulating tubular cell differentiation and the expression of the cancer-causing gene *Pttg1* in the kidney. These findings may provide valuable insights into potential therapeutic strategies for renal cell carcinoma resulting from *SMARCA4* deficiency.

## Introduction

Kidney failure is a devastating health condition and ranks among the top causes of death worldwide. In the United States, more than 37 million adults (15%) are living with chronic kidney disease (CKD), a condition that can progress to kidney failure, currently affecting nearly 786,000 people (www.cdc.gov/kidneydisease). Therefore, it is critical to gain a comprehensive understanding of the disease mechanisms at various levels of regulation, including gene mutation, epigenetic and microRNA regulation. In most kidney diseases, the nephrons − the structures responsible for filtering waste from the blood to producing concentrated urine − lose their filtering capacity. The nephron is a segmented structure consisting of a glomerulus, proximal tube (PT), the Henle’s loop (HL), distal tubule (DT), and the connecting tubule (CNT), which fuses with the collecting duct (CD) system. Each segment has specialized functions in glucose and solute transport, acid-base balance, and water homeostasis. Therefore, correct segmentation of the nephron is crucial for proper kidney function, and any disruption in this process can lead to disease.

Nephrons develop from Six2^+^ multipotent mesenchymal nephron progenitor cells (NPCs) that differentiate into pretubular aggregates (PTAs) upon induction by ureteric bud (UB) (Park et al., 2012). The PTAs then undergo epithelization to form renal vesicles (RVs), which further develop into comma-shaped bodies (CSBs) and S-shaped bodies (SSBs) through a series of invaginations and elongations in the kidney cortical region. While new nephrons are continuously generated, older ones migrate towards the medulla as their HL elongates. Recent studies have emphasized the critical role of cell-cell signaling from the surrounding mesenchymal interstitium in nephrogenesis (Schedl and Hastie, 2000; Li et al., 2014; Wilson and Little, 2021). It is worth noting that each nephron is composed of over 20 different cell types (Al-Awqati and Oliver, 2002), and the kidney contains multiple distinct interstitial cells (Li et al., 2014; Wilson and Little, 2021). Despite recent advances in large-scale sequencing studies (Adam et al., 2017; Lindstrom et al., 2018; Combes et al., 2019; Hochane et al., 2019), our current understanding of how these different cell types are established and maintained in the kidney remains limited.

Proper cell development is determined by gene expression programs, which are coordinately regulated by cell-cycle regulators, lineage-specific transcription factors (TFs), and chromatin regulators. Previous studies have identified several TFs with regionalized expression in the RV/SSB that are critical for nephron segmentation (El-Dahr et al., 2008; Georgas et al., 2009; Little and McMahon, 2012), but the roles of epigenetic/chromatin regulators in kidney development and disease remain poorly understood. Chromatin remodelers regulate chromatin structure in an ATP-dependent manner to facilitate TF binding and interact with TFs to regulate transcription in an ATP-independent manner (Trotter and Archer, 2008). The SWI/SNF (BAF) chromatin remodeling complexes are involved in virtually every stage of cell proliferation and differentiation, stem cells, and cancer (Ho and Crabtree, 2010; Hargreaves and Crabtree, 2011). *SMARCA4* (BRG1) is the central ATPase of BAF complexes and has been identified as a major tumor suppressor (Davoli et al., 2013; Hodges et al., 2016). Somatic mutations in *SMARCA4* are associated with childhood cancers (Howe et al., 2006; Turnbull et al., 2012; Fu et al., 2017; Jia et al., 2017), including Wilms tumors (4.5% prevalence) (Rakheja et al., 2014). We recently characterized the role of *Smarca4* in Six2^+^ NPCs and demonstrated that it interacts with Eya1 and Six2 to maintain the progenitors (Li et al., 2021). Since Smarca4 is also expressed in PTAs, RVs and SSBs (Basta et al., 2020; Li et al., 2021), this prompted us to examine its potential roles in nephron tubulogenesis by specifically deleting *Smarca4* from PTA-RV-SSB stage using the *Wnt4* ^*Cre*^ (*Wnt4* ^*GFPCre*^) line (Mugford et al., 2009; Chung et al., 2017). *Wnt4* is one of the earliest genes marking the differentiation of nephron precursors from the PTA stage (Stark et al., 1994; Mugford et al., 2009; Shan et al., 2010) and is also expressed in collecting duct-associated stroma (Itaranta et al., 2006; Shan et al., 2010; England et al., 2020). Although *Wnt4* ^*Cre*^ mice have previously been used by multiple groups for gene knockout (KO), the molecular characterization of *Wnt4* ^*Cre*^-labeled cells remains unexplored.

Here, we report that *Smarca4* KO in *Wnt4*-expressing (*Wnt4* ^+^) cells led to defective segmentation and elongation of nephron tubules, resulting in a shortened medulla. Through a combination of clustering, transcriptomic trajectories, and pseudotime analysis, we defined the distinctive composition of *Wnt4* ^*Cre*^-labeled cells and uncovered changes in cellular state/identity induced by *Smarca4* KO. Our analyses demonstrated that in *Smarca4*-deficient kidneys, the expression of the oncogene *Pttg1* was significantly upregulated in *Wnt4* ^*Cre*^-labeled cells, which proliferated excessively and displayed elevated expression of cell cycle regulators and genes involved in extracellular matrix (ECM) and epithelial-to-mesenchymal transition (EMT)/fibrosis. Together, our study has uncovered novel cell types and genes associated with *Wnt4* ^*Cre*^-labeled cells. We have identified the crucial role of Smarca4 in regulating tubular cell differentiation and the expression of the cancer-causing gene *Pttg1* in the kidney. These findings may shed new light on therapeutic strategies for renal cell carcinoma resulting from SWI/SNF complex deficiency.

## Materials and methods

### Mice

*Smarca4* ^*fl*^ (MMRRC stock number 036548-UNC) (Sumi-Ichinose et al., 1997) and *Wnt4* ^*tm3(EGFP/cre)Amc*^ (*Wnt4* ^*Cre*^) (Mugford et al., 2009) and *R26-tdTomato* (Madisen et al., 2010) mice were maintained on a 129/Sv and C57BL/6J mixed background. Mice were bred using timed mating, and noon on the day of vaginal plug detection was considered as E0.5. All animal experiments were performed in accordance with animal care guidelines, and the protocol was approved by the IACUC of the Icahn School of Medicine at Mount Sinai (#06-822).

### Histology, *in situ* hybridization (ISH) and immunostaining

Histologic examinations were performed as described previously (Xu et al., 2014). Dissected kidneys were fixed in 4% paraformaldehyde (PFA), embedded in paraffin, and cut into 6–8 μm sections. Whole-mount or section ISH and immunostaining were performed according to standard procedures. For section ISH, kidneys isolated at specific stages were fixed overnight in 4% PFA at 4°C and cryopreserved in 30% sucrose. Tissues were frozen in OCT (Tissue Tek) and sectioned at 9 μm. Antisense and sense RNA probes were generated using T7 or T3 RNA polymerase according to standard procedures.

### Lectins and primary antibodies

Lectin: PHA-L (Phaseolus vulgaris leucoagglutinin lectin) and LTL (Lotus Tetragonolobus lectin) (FL-1111-2 and FL-1321-2, Vector Laboratories). Primary antibodies: anti-Smarca4/Brg1 (ab110641, Abcam), -Wt1 (sc192, Santa Cruz Biotechnology); -THP (Santa Cruz, sc-271022), -NCC (EMD Millipore, AB3553), –phosphohistone-H3 (EMD Millipore, 06-570), -Tnnt2 (ab209813, Abcam), -*a*SMA (clone 1A4 and A5228, Sigma), -Ncam (sc-1507, Santa Cruz), -Vimentin (ab92547, Abcam), -Pttg1 (HPA045034, Atlas antibodies), -Tyrobp (MBS7127061, MyBiosource), -Foxd1 (MBS9204091, MyBiosource), and -Dlk1 (ab210471, Abcam).

### Single-cell preparation and FACS isolation of single tdTomato^+^ cells

For single-cell isolation, kidneys from *R26-tdTomato*, *Wnt4* ^*Cre/+*^ *;R26-tdTomato* or *Wnt4* ^*Cre/+*^ *;Smarca4* ^*fl/fl*^ *;R26-tdTomato* embryos at E18.5 were collected and then minced into 1–3 mm cubes, followed by dissociation in PBS containing 1 mg/mL Dispase and 0.7 mg/mL collagenase IV for 20 min at 37°C with pipetting up and down samples every 5 min. Then, the digested tissues were filtered with a 40-μm nylon cell strain (BD Falcon). The filtered cell suspension was centrifuged at 200 *g* for 5 min. After removal of the supernatant, the cell pellets were washed with PBS twice to remove fragments and then resuspended in PBS containing 1 mM EDTA. To identify and isolate tdTomato^+^ cells, single-cell suspensions of tdTomato^−^ cells from R26-tdTomato was subjected to fluorescence-activated cell sorting (FACS) to establish gates and regions that can distinguish tdTomato^−^ from tdTomato^+^ populations, and subsequently sort and collect tdTomato^+^ cells. We collected control and mutant tdTomato^+^ cells from three embryos (6 kidneys) from 2 different litters respectively.

### scRNA-seq library preparation

Individually barcoded scRNA-seq libraries were prepared using the Chromium Platform (10x Genomics) with the 3′ gene expression V3 kit, using an input of approximately 10,000 cells. Briefly, Gel-bead in emulsions (GEM) were generated on the sample chip in the Chromium controller. Barcoded cDNA was extracted from the GEMs by post-GEM RT-cleanup and amplified for 12 cycles. Amplified cDNA was fragmented and subjected to end repair, poly A-tailing, adapter ligation, and 10 ×–specific sample indexing following the manufacturer’s protocol. Libraries were quantified using Bioanalyzer (Agilent Technologies) and QuBit (Thermo Fisher Scientific) analysis. Libraries were sequenced in paired-end mode on a NovaSeq Instrument (Illumina) targeting a depth of 5 × 10^4^-1 × 10^5^ reads per cell. Sequencing data were aligned and quantified using the Cell Ranger Single-Cell Software Suite (version 3.0, 10 × Genomics) against the provided mm10 reference genome.

### scRNA-seq data analysis

scRNA-seq raw data were processed using the 10X Genomics Cellranger v3 to obtain gene expression counts of each barcode for the mutant and control, respectively. Cell ranger QC was applied to the count matrices to remove non-cells. Filtered gene and barcode count matrices were used for scRNA-seq analysis with a custom R pipeline centered around the *Seurat* package v4.0.3 (Hao et al., 2021), in addition to other publicly available R packages (see below). To remove potential artifacts due to low-quality cells and lowly expressed genes, cells expressing less than 500 genes and genes detected in less than 5 cells were excluded from the analysis. Gene counts for each cell are normalized by dividing the total counts for that cell and multiplying a scale factor of 10,000. Natural-log transformation is then applied to the expression levels plus 1. Next, the top 5,000 most variable genes were identified using the variance stabilizing transformation method. To integrate the mutant and control samples, 5,000 anchor genes were identified between the two samples. After the integration, expression levels were centered and scaled by subtracting the average expression and dividing their standard deviations for each feature. Principal Component Analysis (PCA) dimensionality reduction was applied to the scaled data using the variable genes to calculate the top 30 principal components (PCs). To visualize the cells at a low dimensionality, non-linear dimensional reduction approaches UMAP (McInnes et al., 2018) and tSNE (van der Maaten and Hinton, 2008) were performed using the PCs as input. To cluster the cells, a shared nearest neighbor (SNN) graph (Jarvis and Patrick, 1973) was constructed by calculating the neighborhood overlap between each cell and its 20 nearest neighbors using the Jaccard index (Jaccard, 1912). A local moving algorithm (Waltman and van Eck, 2013) was then performed on the global SNN graph to optimize a modularity function to determine clusters at a resolution of 0.5. To study the detailed cell type characteristics at high resolutions, the cell subsets of tubule progenitors, proximal tubule and stroma were extracted from the global analysis and re-clustered at resolutions of 0.5, 0.25 and 0.4, respectively.

To identify conserved cell cluster markers, differentially expressed genes (DEGs) were identified between a cluster and the rest cells for mutant and control, respectively, using the Wilcoxon Rank Sum test. *p*-values were adjusted by the Benjamini-Hochberg (BH) procedure (Benjamini and Hochberg, 1995). A gene was identified as a potential conserved marker for a cluster if its adjusted *p*-values were less than 0.05 in both mutant and control. The potential markers were then ranked by average log fold change in mutant and control by descending order. The top 100 markers for each cluster were then manually compared with known kidney cell type markers from the previous kidney studies (Combes et al., 2019; Miao et al., 2021) and the PanglaoDB database (Franzen et al., 2019). Cell clusters were then annotated based on the maximum overlap of the cluster markers and cell type markers.

Heatmaps were plotted with the top 4–6 markers from each cell type. DEGs between mutant and control were identified for each cell type using the DESeq2 (Love et al., 2014). *p*-values were corrected using the BH procedure (Benjamini and Hochberg, 1995).

### Gene set enrichment analysis (GSEA) and gene ontology (GO) enrichment analysis

GSEA was performed using ranked fold change values (mutant over control). The enrichment score was calculated according to the GSEA algorithm (Subramanian et al., 2005). GO enrichment analysis was performed using http://bioinformatics.sdstate.edu/idep (Ge et al., 2018).

### Trajectory analysis

Trajectory analysis was performed on the control cells, mutant cells, and both mutant and control combined using the R package *Monocle 3* v1.0.0 (Cao et al., 2019). For the global analysis, the nephron progenitor cells were set as the root cells. For the stroma subclusters, the CD-associated stroma, nephrogenic stromal progenitors or fibroblast cells were set as root cells. For the endothelial cells, endothelial subtype 2 was set as root.

### Reverse transcription and real-time PCR (RT-qPCR)

FACS-purified TdTomato^+^ cells from *Wnt4* ^*Cre/+*^ control and *Smarca4* ^*cKO/cKO*^ kidneys collected from E17.5 kidneys were used for total RNA extraction using Trizol Reagents (15596026, Invitrogen). 0.5–1 μg of total RNAs were treated with RNase-Free DNase I Set (79254, QIAGEN) and then used for reverse transcription using a SuperScript IV Reverse Transcriptase (18090010, Thermo Fisher Scientific) for first-Strand cDNA Synthesis. Real-time PCR was performed using iQ SYBR Green Master Mix (4309155, Applied Biosystems). Expression levels of each transcript were normalized using β-actin as an internal control. Each set of experiments was repeated three times, and the DDCT relative quantification method was used to evaluate quantitative variation.

Oligos used for qPCR: Pttg1-froward 5′-GCA​GTG​GGT​GAA​GTT​GAA​CAC-3′ and Pttg1-reverse 5′-CAG​TGG​TTG​ACA​AGT​TAC​TGT-3'; β-actin-forward 5′-CAT​TGT​TAC​CAA​CTG​GGA​CGA-3′ and β-actin-reverse 5′-GAA​GGT​CTC​AAA​CAT​GAT​CTG-3'.

### Statistics

PH3^+^ cells were counted from sections with clear cortical UB, PTA and SSB and medullary structures. PH3^+^ cells were counted in cortical regions around 32 UBs, including peripheral nephrogenic stroma and ventral PTAs, as well as 32 SSBs per kidney and medullary area 20 × 0.01 mm^2^ per kidney, respectively, and 3 kidneys at E18.5 were counted. Values represent the average number of PH3^+^ cells (±standard deviations) per UB-PTA-SSB for the cortical or 0.01 mm^2^ for the medullary area (section thickness is 6 μm).

TUNEL^+^ cells from 10 sections with clear cortical and medullary structures per E18.5 kidney and 3 kidneys were counted from the medullary region extending into the deep cortical region, respectively. Values represent the average number of TUNEL^+^ cells (±standard deviations) per section (6 μm).

Two-tailed Student’s *t*-test was used for statistical analysis. A value was considered statistically significant if *p* < 0.05.

### Spatial calibration

The area of the *Dll1* ^+^ domain was measured on sections for spatial calibration using ImageJ software (NIH). The area of the *Dll1* ^+^ domain from 18 SSBs (8 sections per kidney and 3 kidneys for each sample) was measured. Two-tailed Student’s *t*-test was used for statistical analysis.

## Results

### *Smarca4* deletion in *Wnt4*-expressing cells leads to severe tubular defects and shortened medulla

**FIGURE 1:**
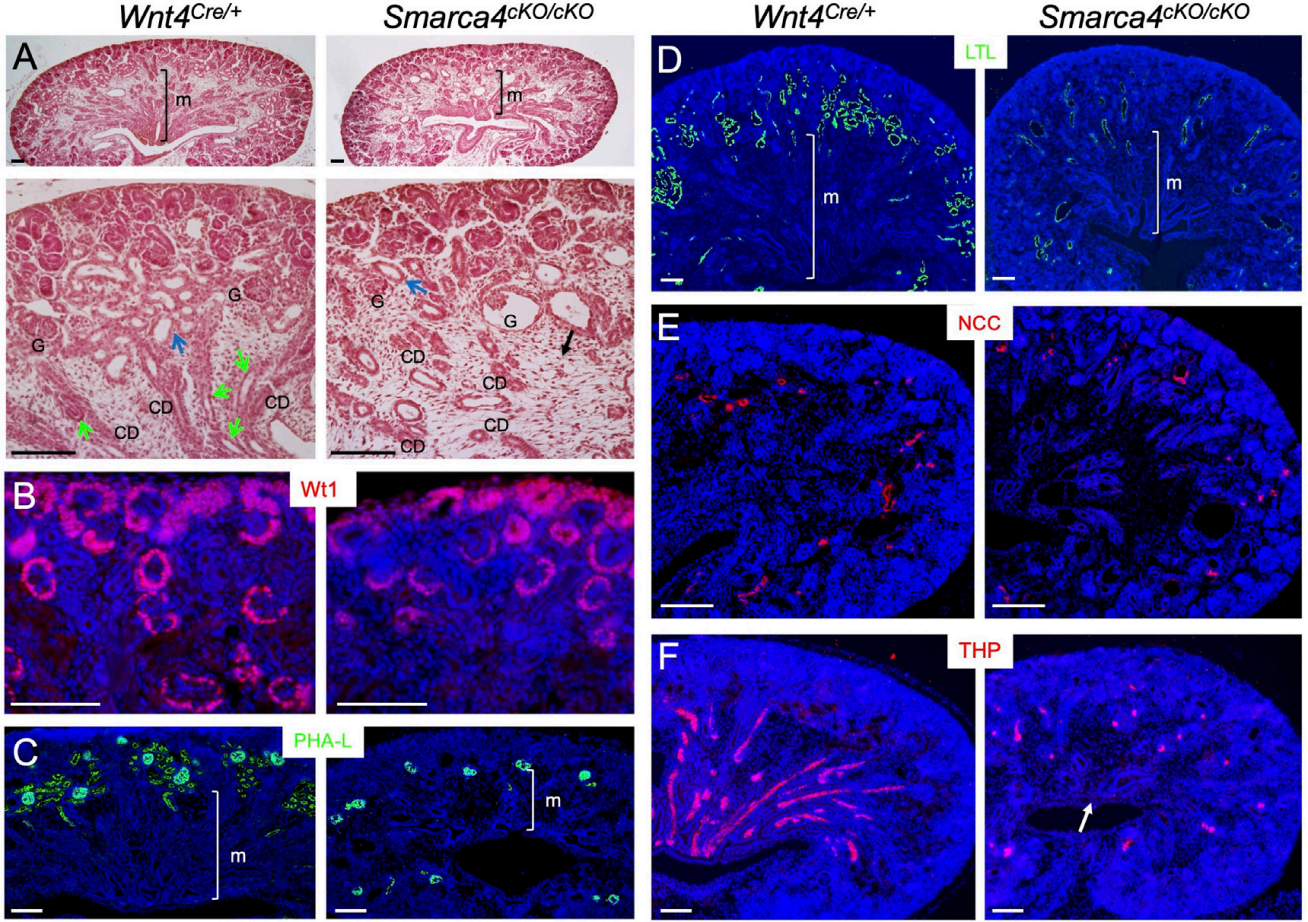
Conditional deletion of *Smarca4* in *Wnt4*-expressing cells leads to abnormal nephron tubule formation. **(A)** H&E-stained mouse kidneys at E18.5 in *Wnt4* ^*Cre/+*^ and *Smarca4* ^*cKO/cKO*^ littermates. The lower panels are a higher magnification of the upper panels. Green arrows indicate Henle’s loop, and blue arrows indicate nephron tubules. The black arrow indicates interstitial mesenchyme in the mutant. Most glomeruli (G) appear normal, but some are cystic. **(B)** Immunostaining for Wt1 (nephron progenitor, PTA/RV and podocyte). **(C,D)** Lectin staining with PHA-L (proximal tubule and podocyte) and LTL (proximal renal tubule). **(E,F)** Immunostaining for NCC (distal renal tubule) and THP (Henle’s loop) in control and mutant kidneys at E18.0. Arrow pints to the reduction of THP^+^ structures in the medullary region of the mutant kidney. abb.: CD, collecting duct; G, glomerulus; M, medullary region. Scale bars: 100 μm.

To investigate whether Smarca4 expression in nephron primordia is required for tubulogenesis, we crossed *Wnt4* ^*Cre/+*^ mice with *Smarca4* ^*fl/fl*^ to delete *Smarca4* in *Wnt4*-expressing (*Wnt4* ^+^) precursors, resulting in the conditional *Smarca4* KO mice (*Smarca4*cKO). Immunostaining confirmed *Smarca4* depletion in CSBs/SSBs of *Smarca4*cKO kidneys (Supplementary Figure S1A). The mutant kidneys at E18.5 were smaller, with a length approximately 24.9% ± 1.6% shorter than that of control littermates (Supplementary Figure S1B; *n* = 8 and *p* = 0.0225). Histological analysis revealed a significantly shortened medullary region with reduced epithelial tubules and increased interstitium (Figure 1A). Quantification of glomerular numbers showed that the number of glomeruli in *Smarca4*cKO was 58.4% ± 1.5% of that in *Wnt4* ^*Cre/+*^ littermates.

Next, we stained kidney sections with antibodies or lectins to label different tubular segments. Wt1 defines podocyte identity by activating other TFs (Dong et al., 2015). As shown by anti-Wt1 immunostaining (Figure 1B), it is expressed in the podocyte primordium of the proximal RV/SSB and in developing podocytes. In *Smarca4*cKO kidneys, Wt1 expression in these structures was preserved. The mutant podocytes were also marked by lectin PHA-L (Figure 1C). Remarkably, however, PHA-L-marked PTs in the cortex were markedly reduced (Figure 1C). Similarly, lectin LTL-labeled PTs, especially the proximal convoluted tubules (PCT), were significantly decreased (Figure 1D). While both distal convoluted tubules (DCT) marked by NCC (NaCl cotransporter) (Figure 1E) and HLs labeled with THP (Figure 1F) were detectable, the THP^+^ HLs were found in similar locations as the PTs or DCTs and failed to reach the innermost region of the kidney. These results indicate that *Smarca4* is essential for proper tubulogenesis.

### Single-cell sequencing identifies previously unknown cell populations linked to *Wnt4* ^*Cre*^-labeled cells

**FIGURE 2:**
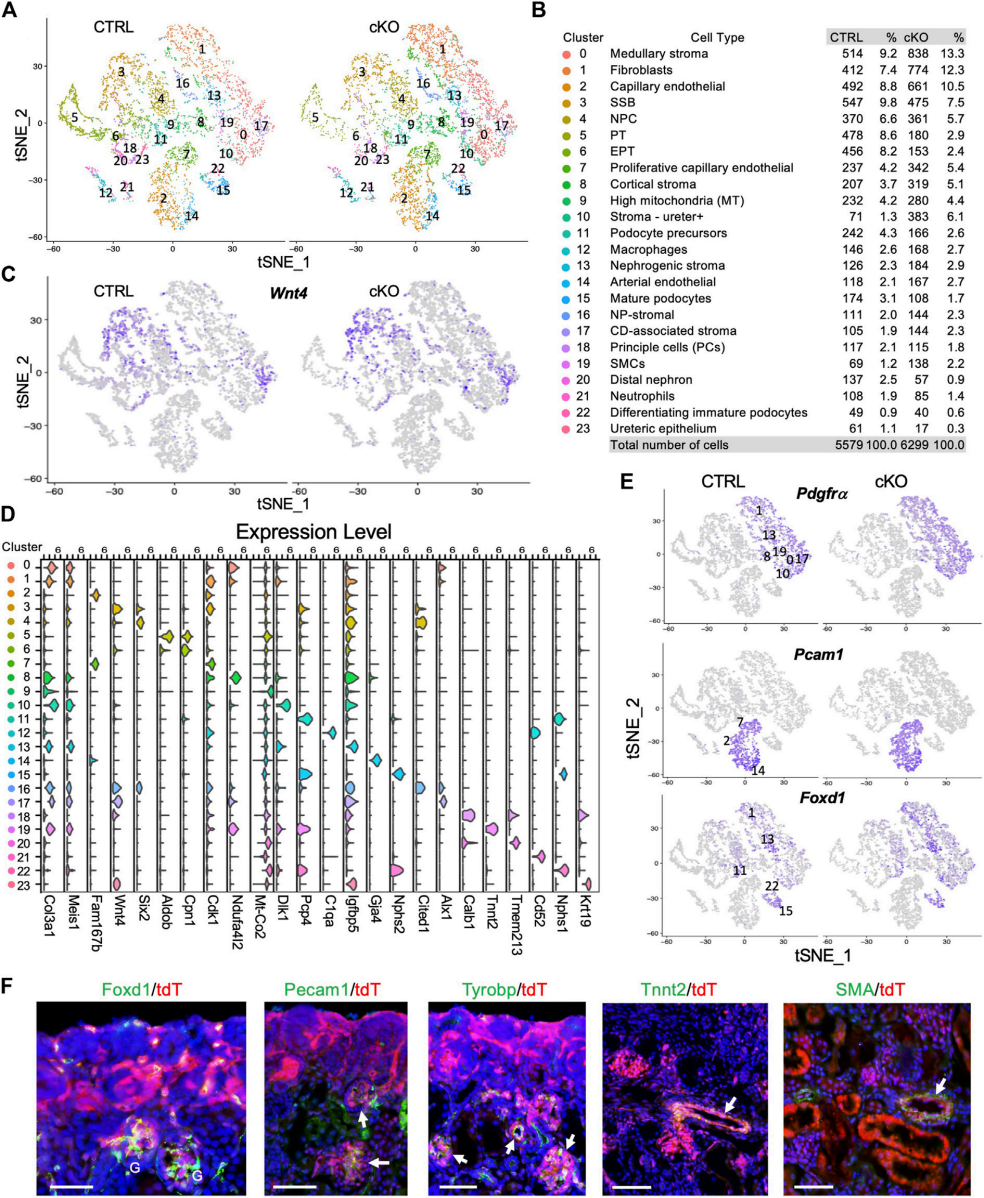
scRNA-seq delineates distinct types of cells derived from *Wnt4*-expressing cells and the effects of *Smarca4* deficiency in these cells. **(A)** Unsupervised clustering demonstrates 24 distinct cell types shown in a tSNE plot of *Wnt4* ^*Cre*^-labled cells of control and *Smarca4* ^*cKO/cKO*^. **(B)** Percentages of assigned cell types are summarized in the right panel. NP, nephron progenitor; SSB, S-shaped body; NPC, nephron progenitor cell; PT, proximal tubule; EPT, early PT, SMCs, smooth muscle cells; CD-associated stroma, collecting duct-associated stroma; **(C)** tSNE plots showing restricted *Wnt4* expression. **(D)** Violin plot showing the expression levels of representative marker genes across the 24 main clusters. The *y*-axis shows the long-scale normalized read count. **(E)** tSNE plots showing *Pdgfrα* in stroma cells, *Pecam1* expression in endothelial cells and *Foxd1* expression in stroma of nephrogenic, cortical and podocytes. **(F)** Immunostaining for Foxd1, Pecam1, Tyrobp, Tnnt2 and SMA showing colocalization with tdTomato^+^ cells in *Wnt4* ^*Cre/+*^ *;R26-tdTomato* kidneys (arrows). Scale bar: 60 μm.

Since the knowledge of *Wnt4* ^+^ cells and the different cell types they may differentiate into is sparse, we set out to comprehensively define the cellular state of *Wnt4* ^*Cre*^ *-*labeled cells and further investigate the requirement of *Smarca4* during nephron differentiation at a single-cell resolution. We performed scRNA-seq analysis on FACS-purified fresh tdTomato^+^ cells from control or *Smarca4*cKO kidneys at ∼ E18.0-18.5 (harvested 7 a.m.) (Supplementary Figure S2A). After removing low-quality cells, we obtained transcriptomes from 5,579 control and 6,299 *Smarca4*cKO cells, respectively. Unsupervised analysis identified 11 major cell types (Supplementary Figure S2B, C), which can be divided into 24 distinct cell clusters (CS) (Figures 2A, B). All cell types largely overlapped between the control and mutant.

Next, we conducted differentially expressed gene (DEG) analysis between each cluster and the rest of the clusters in the control to further characterize *Wnt4* ^*Cre*^ *-*labeled kidney cell types and determine the key marker genes for each cell type (full lists of cluster markers are provided in Supplementary File S1). SSB (CS3) and CD-associated stroma (CS17) exhibited high levels of *Wnt4* expression (Figures 2C, D), in agreement with previous ISH findings (England et al., 2020). Consistent with a previous report indicating the re-entry of *Wnt4* ^+^ PTA cells into the NPCs (Lawlor et al., 2019), we identified *Six2* ^+^ *Cited1* ^+^ NPCs (CS4) (Figure 2D; Supplementary Figure S2D). Clusters corresponding to major nephron segments (CS5,6,20) and podocytes (podocyte precursors CS11–high levels of *Pcp4*, mature podocytes CS15–high levels of *Nphs1/Nphs2*, and immature podocytes CS22–high levels of *Nphs1* but low *Nphs2*) (Supplementary Figure S2D) were identified. Notably, a large proportion of *Wnt4* ^*Cre*^-labled cells were stromal cells (*Col3a1/Pdgfrα*, Figures 2D, E) and *Pecam1* ^+^ endothelial cells (ECs) (Figure 2E). The ECs could be separated into capillary (CS2– *Rgcc* ^*+*^ *Fabp4* ^*+*^ *Fabp5* ^*+*^ but low *Cdk1*, Supplementary Figure S2E), proliferative capillary (CS7–*Rgcc* ^*+*^ *Fabp4* ^*+*^ *Fabp5* ^*+*^ but high *Cdk1,* Supplementary Figure S2E), and arterial (CS14–*Gja4* ^*+*^) (Figure 2D; Supplementary Figure S2E; Supplementary File S1). We also identified a small population of SMCs (CS19–*Tnnt2* ^*+*^ *,* Figure 2D), 2 types of immune cells (*Tyrobp* ^+^, Supplementary Figure S2E)—macrophages (CS12–*C1qa* ^*+*^) and neutrophils (CS21–*Cd52* ^+^) (Figure 2D), principal cells (CS18 –*Calb1* ^+^) and a small population of ureteric epithelial cells (CS23–*Krt19* ^+^ *Igfbp5* ^+^) (Figure 2D). We noticed higher mitochondrial levels in cluster 9 (Figure 2D), we, therefore, excluded this cluster for further analysis.

*Wnt4* is expressed in stromal cells around the ureteric tree in the medullary regions (England et al., 2020) and plays a critical role in the SMC fate commitment in the medullary stroma (Itaranta et al., 2006). These *Wnt4* ^+^ cells were thought to be a transient stromal cell population because previous studies did not detect *Wnt4* ^*Cre*^-labeled SMCs, endothelia or macrophages/neutrophils by expression studies with cell type-specific markers on kidney sections (Shan et al., 2010). However, our deep sequencing analysis clarified the presence of these cell populations as well as cortical stroma and nephrogenic stroma (Figures 2B, E). To validate these genomic findings, we visualized the expression of Foxd1, Pecam1, Tyrobp, and Tnnt2/SMA (smooth muscle actin) by immunostaining and observed that tdTomato^+^ cells overlapped with Foxd1^+^ nephrogenic/cortical stromal cells and podocytes, Tnnt2^+^/SMA^+^ SMCs, Pecam1^+^ ECs, and Tyrobp^+^ immune cells (Figure 2F). Thus, this analysis revealed the heterogeneity of *Wnt4* ^*Cre*^-labeled cells in the mouse kidney, including previously unknown cell types.

### Effects of *Smarca4* deficiency on *Wnt4* ^Cre^-labeled cells: changes in cellular state and developmental trajectory

To determine the effect of *Smarca4* loss on *Wnt4* ^*Cre*^-labeled cells, we compared datasets between the control and *Smarca4*cKO. Although all cell types overlapped between the control and mutant, we observed a significant reduction in the number of podocytes and tubular cells in the mutant (Supplementary Figure S2C). This finding aligns with the results from the phenotypic analyses (Figure 1). Conversely, the mutant exhibited an increase in the proportion of ECs, SMCs and stromal cells (Supplementary Figure S2C). The DEG analysis conducted between the control and mutant groups (Supplementary File S2) revealed a widespread upregulation of the housekeeping ribosome gene *Rps18* (Gm10260), the actin-binding protein *Tpm3-rs7,* and the nucleosome *Hist1h2ap* (Supplementary Figure S3A). These genes are tightly associated with cell proliferation and growth in high-protein synthesis-demanding cells (Kusnadi et al., 2015; Gaviraghi et al., 2019; Ferreira et al., 2020) and are frequently dysregulated in highly proliferative cancer cells (Catez et al., 2019; Gaviraghi et al., 2019; Ferreira et al., 2020). Therefore, the upregulation of these genes likely indicates changes in the cellular state caused by *Smarca4* deficiency.

To further investigate the changes in the cellular state in the mutant, we employed pseudotime ordering analysis (Haghverdi et al., 2016) to evaluate cellular developmental trajectories. Consistent with the upregulation of *Rps18* (Gm10260), *Tpm3-rs7,* and *Hist1h2ap,* this analysis indicated an overall increase in proliferative progenitors in the mutant. For example, three EC clusters shared a common progenitor residing in the proliferating capillary progenitors, but *Smarca4*cKO produced more proliferative progenitors (Supplementary Figure S2F). The analysis of nephron tubule differentiation revealed that the mutant produced more precursors that continued along the nephron differentiation path, and the differentiation status of podocytes appeared different from control cells (Supplementary Figure S3B). These findings further suggest that *Smarca4* deficiency alters the cellular state and fate (e.g., cell proliferation and differentiation). Together, these results provide important insights into the impact of *Smarca4* on the developmental trajectory of *Wnt4* ^*Cre*^-labeled cells, which could have implications for kidney development and disease.

### Identification of *Wnt4* ^*Cre*^ *-*labeled stromal cell populations and enhanced *Pttg1* expression in *Smarca4*cKO

**FIGURE 3:**
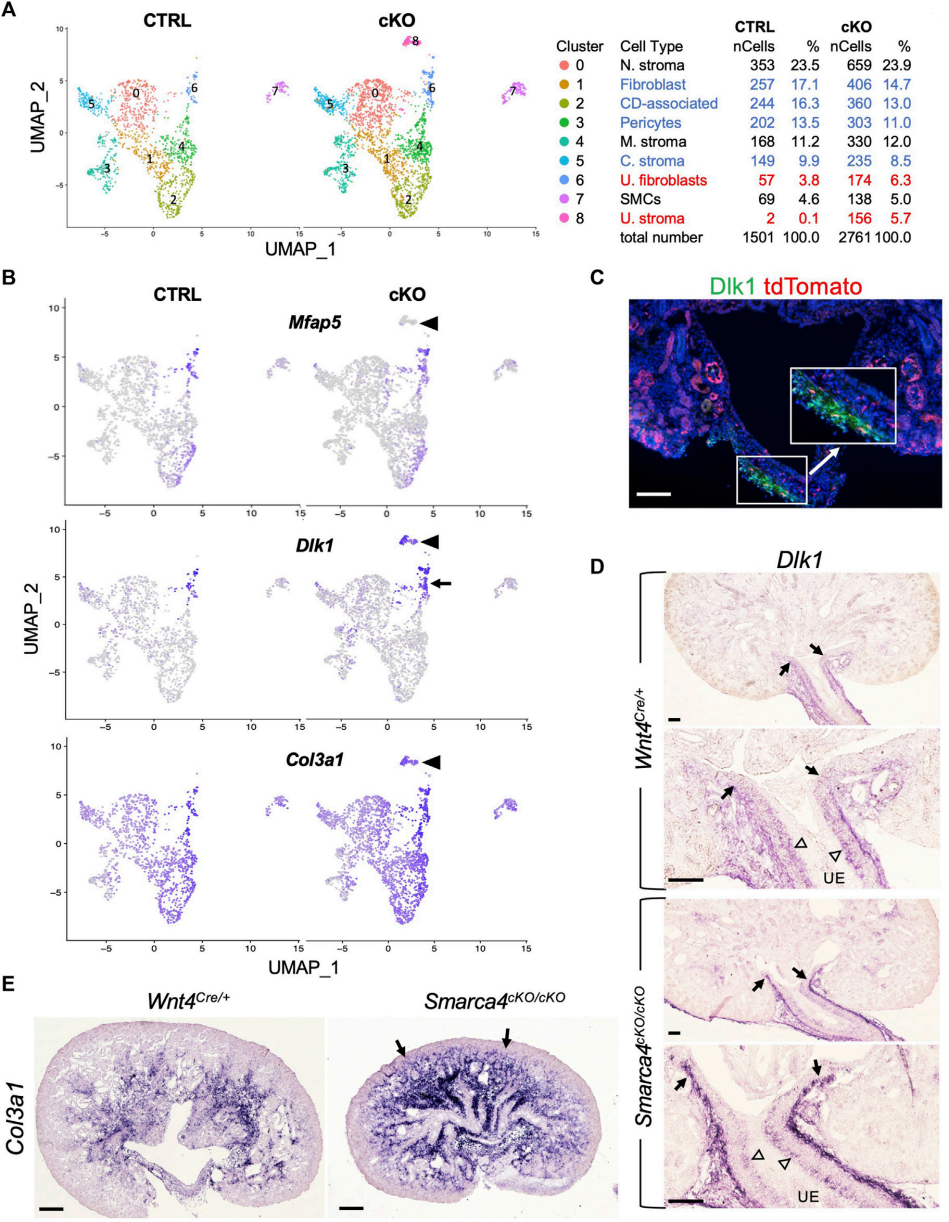
scRNA-seq analysis reveals distinct types of stromal cells derived from *Wnt4*-expressing cells and upregulation of *Dlk1* and *Col3a1* in *Smarca4* ^*cKO/cKO*^. **(A)** The UMAP plot of *Wnt4* ^*Cre*^-labeled stromal populations and SMCs of control and *Smarca4* ^*cKO/cKO*^. Percentages of assigned cell types are summarized in the right panel. Colored fonts indicate representative clusters of increased (red) or decreased (blue) cell numbers in the mutant. **(B)** The UMAP showing *Mfap5, Dlk1* and *Col3a1* expression. Arrowheads point to a ureteric stoma population (*Mfap5* ^-^) that emerged only in the mutant. Arrow indicates the upregulation of *Dlk1* in the mutant. **(C)** Immunostaining for Dlk1 (green) on kidney section of *Wnt4* ^*Cre/+*^ *;tdTomato*. Boxed area is also shown in higher magnification (arrow). **(D)** ISH on kidney sections showing *Dlk1* expression in ureteral stroma in the outer layer (arrows) and some in the inner layer (open arrowheads) adjacent to the ureteric epithelium (UE) in the ureteropelvic kidney region and upregulation in the outer mesenchymal layer. **(E)** ISH on kidney sections at E17.5 showing upregulation of *Col3a1* in *Smarca4*cKO. Arrows indicate the extension of *Col3a1* expression to the cortical nephron primordium areas. Scale bars: 100 μm in **(C,D)** and 200 μm in **(E)**.

**FIGURE 4:**
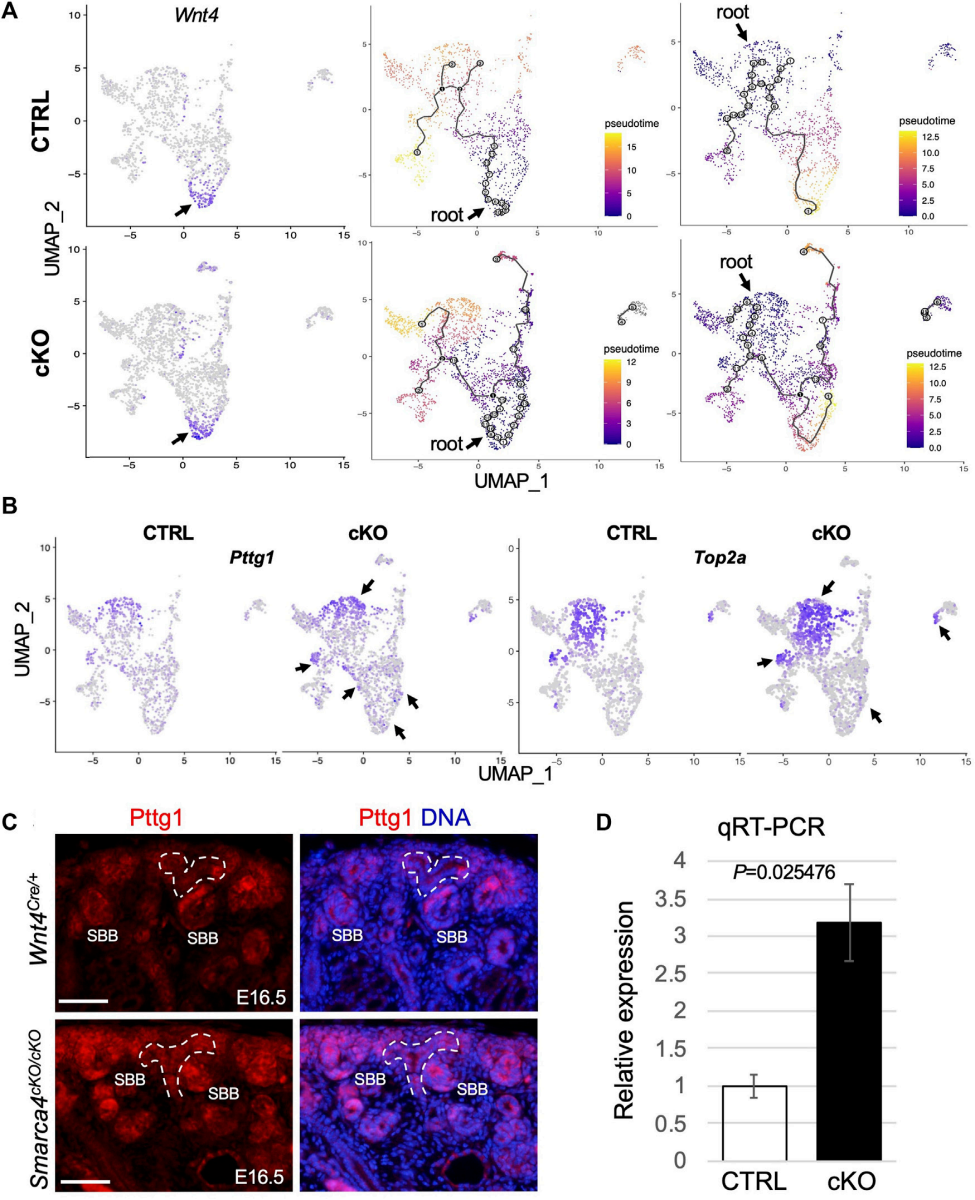
*Smarca4* KO leads to alterations in the developmental trajectories of stromal cells and increased *Pttg1* expression. **(A)** UMAP showing strong *Wnt4* expression in CD-associated stroma (arrows) and in some nephric and ureteric stromal cells and stromal cell differentiation trajectory from *Wnt4*-expressing CD-associated stromal progenitors towards cortical and ureteric stroma (middle panels) or from nephrogenic stromal progenitors towards other stroma cells (right panels). Cells are colored by pseudotime. The circles with numbers denote special points within the graph. Each leaf, denoted by light gray circles, corresponds to a different outcome (i.e., cell fate) of the trajectory. Black circles indicate branch nodes where cells can travel to one of several outcomes. The numbers within the circles are provided for reference purposes only. Note the lack of black circles in the control using nephrogenic stroma as the root. **(B)** UMAP representation of increased *Pttg1* and *Top2a* transcription in stromal cells in *Smarca4* ^*cKO/cKO*^ (arrows). **(C)** Immunofluorescence staining for Pttg1 showing increased levels of Pttg1 in *Smarca4* ^*cKO/cKO*^ kidneys at E16.5. Arrows point to a tubular structure in the mutant. Branching ureteric buds are outlined by dashed lines. Abb.: SBB, S-shaped body. Scale bar: 50 μm. **(D)** Quantitative RT-PCR of FACS-purified tdTomato^+^ cells from control and *Smarca4*cKO kidneys. qPCR was performed in triplicate and repeated three times.

Our findings reveal that *Wnt4* ^*Cre*^-labeled cells comprise multiple distinct stromal populations that are enriched in *Smarca4*cKO kidneys. However, the origin and regulatory networks that control the development of distinct stromal cells in the kidney are poorly defined. Therefore, we extracted stromal populations along with SMCs and subclustered them into 9 subpopulations (Figure 3A; Supplementary File S3). Heatmap analysis of the top 4 marker genes (Supplementary Figure S4A) revealed differences between ureteric stromal cells in control and *Smarca4*cKO kidneys, with a subpopulation in the mutant lacking the expression of *Mfap5* (Figure 3B) and *Tbx18* (Supplementary Figure S4B) but expressing *Wnt4* (Figure 4A). These cells also expressed *Dlk1* (Figure 3B; Supplementary Figure S4A), *Col3a* (Figure 3B), and *Itm2a* (Supplementary Figures S4A, B), as well as all other top DEGs expressed in *Mfap5* ^+^ *Tbx18* ^+^ ureteric stroma cells (Supplementary File S3).

To confirm this finding, we conducted immunostaining for Dlk1 and observed its expression in the stroma at the ureteropelvic junction and in the ureter, which co-localized with tdTomato^+^ cells (Figure 3C). Furthermore, we performed ISH and found that *Dlk1* transcripts were weakly expressed in the ureteric stroma at the ureteropelvic region, but were substantially upregulated in the outer layer in the mutant (Figure 3D). We also observed some *Dlk1* expression in the inner stromal cells, which was not significantly increased in the mutant (open arrowheads, Figure 3D). Based on previous lineage tracing studies, the outer adventitial cells and the inner lamina propria fibroblasts adjacent to the ureteric epithelium derive from *Tbx18* ^+^ precursors (Bohnenpoll et al., 2017). Therefore, some *Tbx18* ^+^ cells in the *Dlk1* ^+^ *Mfap5* ^+^ subgroup may represent precursors or intermediate cells, while the *Dlk1* ^high^ outer layer of the ureteric stroma in the mutant may be mixed with some *Dlk1* ^+^ *Mfap5* ^-^ cells. As *Mfap5* ^+^ cells (Figure 3B) were negative for *Wnt4* (Figure 4A), increased Wnt4 signaling in some ureteric stromal cells may lead to changes in the expression of specific genes, such as the downregulation or inhibition of *Mfap5* expression. We also confirmed increased *Col3a1* transcripts in the mutant (Figure 3E), which is consistent with the upregulation of *Col3a1* detected by scRNA-seq (Supplementary Figure S3A).

To gain insight into the lineage relationships of different *Wnt4* ^*Cre*^-labeled stromal populations, we conducted pseudotime analyses. We selected *Wnt4* ^high^ CD-associated stromal cells (Figure 4A) as the “root” for our model in which *Wnt4* ^+^ “progenitors” expand and produce a group of progenitors that differentiate into fibroblasts. These fibroblasts then give rise to precursors in the nephrogenic stroma, which further branches and differentiate into pericytes (Figure 4A). Interestingly, we found no direct lineage connection between the medullary stroma or ureteric fibroblasts and the CD-associated stromal cells, suggesting that these cells may originate from different progenitor cells. However, in *Smarca4cKO*, the CD-associated *Wnt4* ^high^ progenitors not only produce more cycling/proliferating cells but also generate fibroblasts that can differentiate into the mutant medullary and ureteric stroma. We observed similar linear relationships in a model where the nephrogenic stroma (Figure 4A) or fibroblasts (Supplementary Figure S4C) were chosen as the “root”. However, when using fibroblasts as the root, more cells showed gene expression changes along the trajectory (grey circles, Supplementary Figure S4C). In contrast, none of these models exhibited a direct linear relationship with the cortical stroma or smooth muscle cells (SMCs), indicating that these cell types likely originate from different progenitor cells. Overall, these analyses underscore the intricate development of *Wnt4* ^*Cre*^-labeled stromal cells and reveal increased cell proliferation and altered developmental trajectories caused by *Smarca4* deficiency.

After applying stringent cutoffs of adjusted *p* < 0.01 and fold-change >1.5, the DEG analysis identified only a few upregulated genes in *Smarca4*cKO. Of these genes, *Pttg1* was globally upregulated, particularly in nephrogenic stromal cells, pericytes and some CD-associated stroma (Figure 4B; Supplementary File S4). *Pttg1* plays a crucial role in sister chromatid segregation during mitosis, and its upregulation is associated with various types of tumors (Pei and Melmed, 1997; Itaranta et al., 2006; Chen et al., 2020). We also detected a similar upregulation of *Top2a* (Figure 4B), a proliferation marker associated with tumor grade and Ki67 index (Supplementary Figure S4B). Next, we used two independent methods to confirm the upregulation of *Pttg1* in the mutant. First, we immunostained for Pttg1 at the earlier stages of E16.5-17.5 and found that the levels of Pttg1 were increased in the mutant kidneys at these stages, especially in the cortical and PTA-SSB regions (Figure 4C). Second, we performed reverse-transcription and real-time PCR (RT-qPCR) using RNA prepared from freshly FACS-purified tdTomato^+^ cells from E16.5 kidneys and found that *Pttg1* expression levels in *Smarca4*cKO were ∼3.2-fold higher than in control cells (Figure 4D). Altogether, our findings demonstrate that *Smarca4* deficiency induces *Pttg1* upregulation in *Wnt4* ^*Cre*^-labeled cells.

### *Smarca4* KO disrupts early patterning of SSBs

**FIGURE 5:**
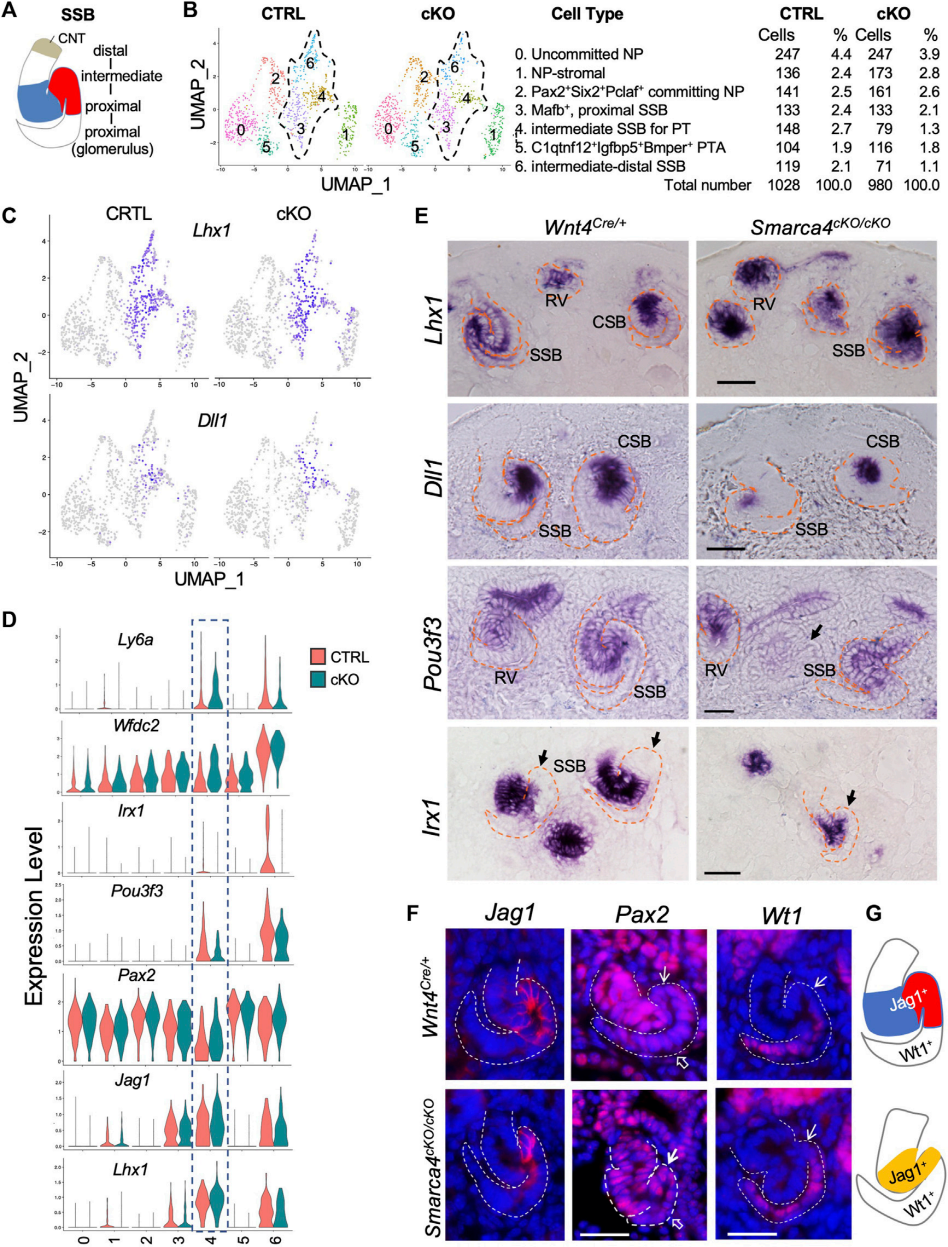
Early patterning of SSB was disrupted in *Smarca4-*deficient kidneys. **(A)** Schematic drawing of distinct nephron territories in SSB. CNT, connecting tubule segment. **(B)** UMAP plots showing 7 clusters of nephron precursors derived from *Wnt4* ^*Cre*^-labeled cells in control and *Smarca4* ^*cKO/cKO*^. Percentages of assigned cell types are summarized in the right panel. **(C)** UMAP plots showing *Lhx1* and *Dll1* expression in the SSB clusters. **(D)** Violin plots showing the levels of *Lhx1, Jag1, Pax2, Pou3f3, Irx1, Wfdc2* and *Ly6a* expression in each cluster. Boxed SSB-PT cluster 4 showing increased *Pax2*, *Wfdc2* and *Ly6a* in the mutant but no significant changes in the expression levels of *Lhx1* and *Jag1*, while *Pou3f3* and *Irx1* expression levels were reduced in the mutant. **(E)** *In situ* hybridization for *Lhx1*, *Dll1*, *Pou3f3*, and *Irx1* on kidney sections of E15.0 embryos (harvested around 5 p.m.). Note reduced *Pou3f3* expression in some SSB in the mutant (arrow), and *Irx1*-negative proximal tubule primordium in control (arrows) and the absence of this region in the mutant (arrow). **(F)** Immunostaining for Jag1, Pax2, and Wt1 (podocyte) at E15.5. Arrows point to the PT territory and open arrows point to the podocyte regions. **(G)** Schematic summary of SSB Jag1^+^ domain in control and mutant. Abb.: CSB and SSB, comma- and S-shaped body; RV, renal vesicle. Scale bars: 30 μm.

We next investigated whether *Smarca4* loss resulted in changes in the expression of genes involved in early SSB segmentation and upregulation of *Pttg1* in nephron precursor cells. The SSB is patterned into a proximal segment—Bowman’s capsule and visceral podocyte—derived from the proximal domain of the polarized RV, a distal RV-derived part that further subdivides into the future PT, HL, and DT segments followed by CNT fused with CD (Figure 5A). Subclustering analysis of nephron precursor cells (CS3, 4, 16) revealed 7 distinct populations: uncommitted NP, NP-stromal, committing NP, PTA-RV, proximal SSB for podocyte, intermediate SSB for PT, and intermediate-distal SSB for HL and DT (Figure 5B; Supplementary File S5).

We next analyzed changes in the expression of genes involved in early patterning of SSB, including *Lhx1*, *Notch* signaling, and other TFs, which was verified by ISH or immunostaining. *Lhx1* is required for initial RV polarization and expressed in distal RV and intermediate-distal SSBs that form PT, HL and DT (Kobayashi et al., 2005) (Figures 5C–E). The Notch ligand Dll1 further specifies the *Lhx1* ^+^ domain into the PT fate, as *Dll1* hypomorphic mice have severely reduced nephron numbers with loss of PT (Cheng et al., 2007). *Dll1* is expressed in the distal RV, and its expression persists in the middle region of the SSB where PT and HL are generated (Figures 5C, E). Jag1, another Notch ligand, is highly expressed in the intermediate SSB for PT and HL (Figures 5D, F). The DEG analysis did not detect significant changes in expression levels of these three genes (Figures 5C, D; Supplementary File S6). Consistent with this, no obvious changes in the expression levels of these genes were observed by ISH (Figure 5E) or immunostaining (Figure 5F), although *Smarca4*cKO kidneys at ∼ E15.0 were smaller (Supplementary Figure S5A), and *Dll1*-marked region (Figure 5E) or Jag1^+^ domain (Figures 5F, G) was reduced in size. We measured and calibrated the area of the *Dll1* ^+^ domain and found that the *Dll1*-marked area was ∼53.22% ± 1.8% smaller than the control (*n* = 18, *p* = 0.02808). In contrast, *Notch1* expression was downregulated in *Smarca4*cKO SSB-PT territory (Supplementary Figure S5B; Supplementary File S6), while *Notch2* did not show significance changes (Supplementary Figure S5B; Supplementary File S6). Pax2 also showed segmented expression in the SSB, with lower expression levels in the future PT regions (Figures 5D, F). However, *Pax2* expression levels in the mutant PT regions were higher than in the control (Figures 5D, F; Supplementary File S6). Notably, the HL/DT-specific genes, such as *Wfdc2* and *Ly6a* were also upregulated in the mutant prospective PT region (Figure 5D).

*Pou3f3* plays a crucial role in determining the fate of intermediate and distal nephrons (Nakai et al., 2003). It is expressed in the RV and broader regions covering the mid-distal SSB (Figure 5E, Supplementary Figure S5A). However, its expression was reduced in some mutant SSBs (arrow, Figure 5E), which is consistent with the findings from the scRNA-seq analysis (Figure 5D). We also investigated the expression of Irx genes, which are essential for the formation of the intermediate region of the nephron (Reggiani et al., 2007). *Irx1* expression begins at the CSB stage and is confined to the intermediate region destined to become HL (Figures 5D, E). In *Smarca4*cKO kidneys, the number of *Irx1*-labeled nephron primordia was significantly decreased (Supplementary Figure S5A), and the SSBs had smaller *Irx1* ^*+*^ regions and lacked a well-defined *Irx1* ^*-*^ future PT fate (arrows, Figure 5E). Additionally, the intensity of *Irx1* and *Irx2* signals appeared to be reduced in the mutant (Figure 5E; Supplementary Figure S5A). These findings align with the scRNA-seq analysis for *Irx1* (Figure 5D; Supplementary File S6) and *Irx2* (Supplementary File S6). Together, these results indicate that *Smarca4* is necessary for establishing distinct nephron tubular regions during SSB segmentation and extension.

### *Smarca4* loss induces upregulation of *Pttg1* and ECM-related genes in committing nephron precursors accompanied by hyperproliferation

**FIGURE 6:**
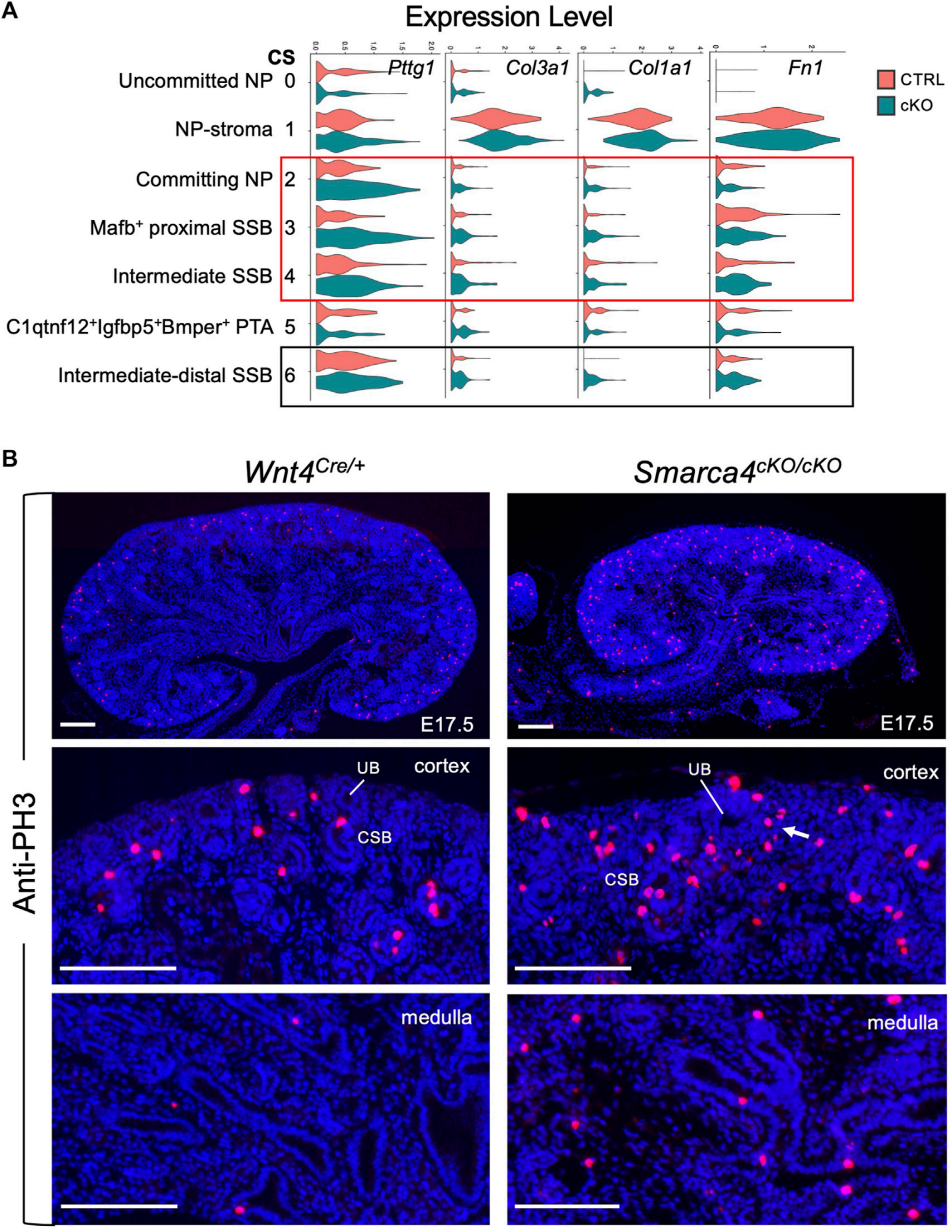
*Smarca4* loss induces upregulation of *Pttg1* and mesenchymal ECM genes in nephron precursor cells as well as increased proliferation. **(A)** Violin plots showing the levels of *Pttg1, Col3a1, Clo1a1* and *Fn1* expression in each cluster. Boxed areas indicate increased *Pttg1* expression in the committing NP and SSB clusters and increased expression of *Col3a1*, *Col1a1* and *Fn1* in the SSB clusters. **(B)** Anti-PH3 immunostaining showing increased PH3^+^ cells in *Smarca4* ^*cKO/cKO*^ kidney. Arrow points to increased PH3^+^ cells in committing NP-PTA area. Quantification (see Methods) revealed that PH3^+^ cells in *Smarca4cKO* were 4.42 ± 0.23- or 7.5 ± 0.35-fold higher in cortex or medulla than controls. Abb.: CSB, comma-shaped body; UB, ureteric bud. Scale bar: 100 μm.

Our analyses also indicated upregulation of *Pttg1* (Figure 6A, Supplementary Figure S5C) and *Top2a* (Supplementary Figure S5C) in the mutant SSB. We also observed elevated expression of ECM markers *Col3a1/Col1a1/Fn1* in the mutant (Figure 6A), indicating a potential progression towards fibrosis or EMT. These findings underscore the critical role of Smarca4 in regulating the expression of *Pttg1* and the cellular state and fate of nephron precursors.

Since Pttg1 is a key regulator of sister chromatid segregation during mitosis and its increased PTTG1 expression is associated with hyperproliferation, we clarified whether *Smarca4* loss also leads to increased proliferation. Immunostaining for anti-phospho-Histone H3 (PH3), a marker for M-phase cells, showed a significant increase in PH3^+^ cells in the cortex and medulla of *Smarca4*cKO kidneys compared to controls (Figure 6B, ∼4.42-fold in cortex and ∼7.5-fold in medulla). This result is consistent with the transcriptomic trajectories showing increased proliferating/cycling cells in the interstitial cells (Figures 4A, B; Supplementary Figure S2F) and nephron precursors (Supplementary Figure S3B) of *Smarca4*cKO kidneys. Given that PTTG1 as an oncogene known to promote cell division and contribute to the development of tumors and metastasis (Luo et al., 2013; Noll et al., 2015), the elevated levels of Pttg1 and increased cell proliferation rate in *Smarca4cKO* may lead to upregulation of ECM-related genes in the precursor cells and limit their ability to differentiate into proper tubular cells.

### *Smarca4* deficiency causes mixed cell identities, increased proliferation and ECM/EMT-associated gene expression in tubular cells

**FIGURE 7:**
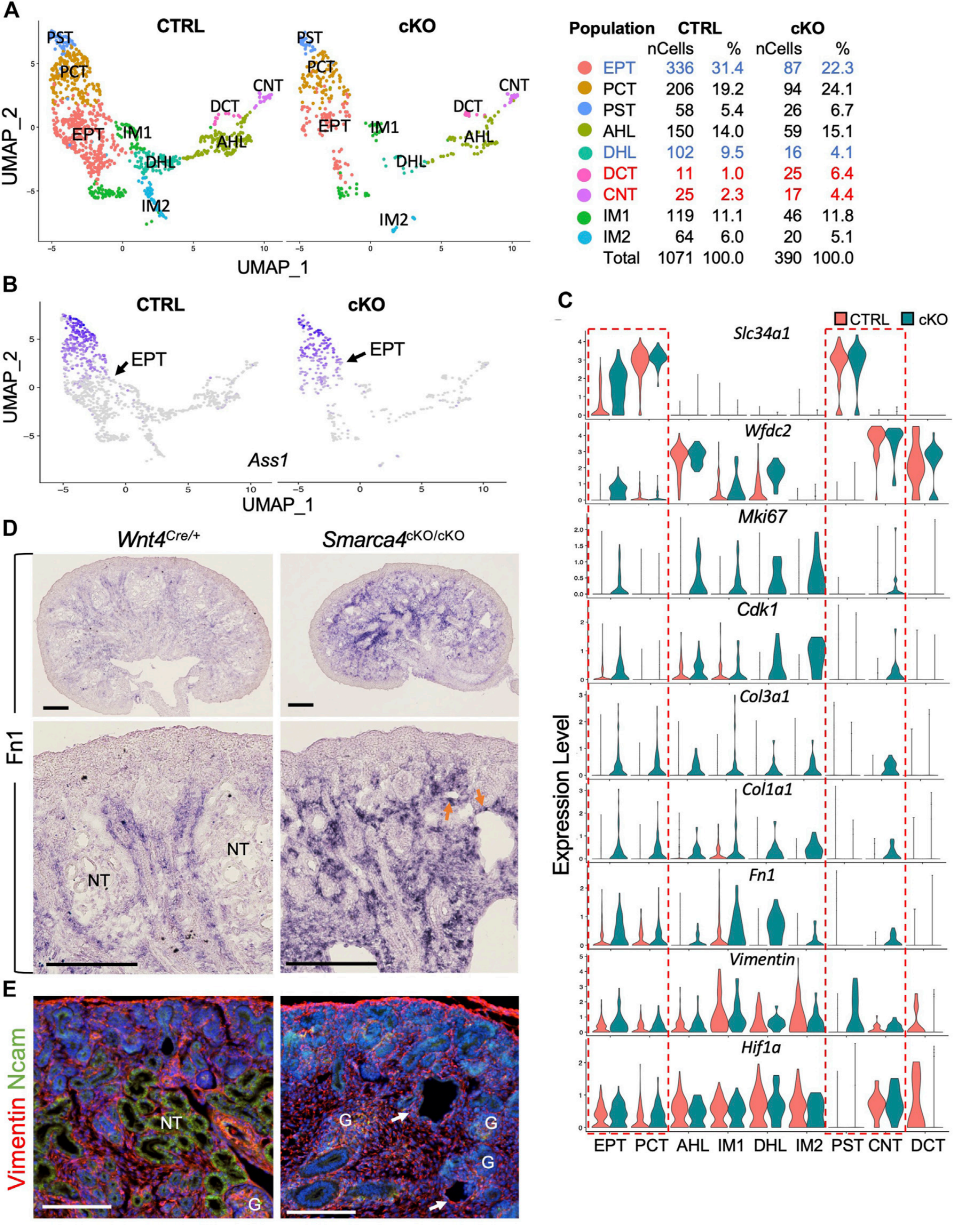
The expression of ECM and EMT markers are upregulated in nephron tubule cells in *Smarca4*cKO. **(A)** UMAP plot showing 9 major types of nephron tubule cells derived from *Wnt4* ^*Cre*^-labeled cells. Percentages of assigned cell types are summarized in the right panel. Colored fonts indicate representative clusters of decreased (blue) or increased (red) cell numbers in the mutant. **(B)** UMAP plot showing the upregulation of *Ass1* expression in the EPT cells (arrows) in *Smarca4*cKO. **(C)** Violin plots showing the expression of *Slc34a1, Wfdc2, Mki67, Cdk1, Col3a1, Col1a1, Fn1, Vimentin* and *Hif1a* in control and *Smarca4*cKO cell clusters. Boxes outlined in the dashed redline indicate increased expression of *Slc34a1* and *Wfdc2* in the mutant EPT and other genes in the mutant EPT, PCT, PST, and CNT. **(D)** *In situ* hybridization on kidney sections at E17.5 showing upregulation and extension of *Fn1* into the cortical region of the mutant kidneys. Arrows indicate *Fn1*-expressing cells in nephron tubular structures. **(E)** Immunostaining for Vimentin (red) and Ncam (green) showing increased Vimentin^+^ cells and the absence of Ncam^+^ epithelial tubules (arrows) in the mutant kidney. Abb.: G, glomerulus; NT, nephron tubule. Scale bar: 300 μm [top panels in **(D)**] and 100 μm [bottom panels in **(D)** and panels in **(E)**].

To further investigate the impact of *Smarca4* deficiency on nephron differentiation, we re-clustered the nephron tubule populations (CS5,6,20) and identified two progenitor-tubule intermediate populations (IM1, IM2) and seven distinct nephron tubule populations, including EPT, PCT, PST (proximal straight tubule), DCT and CNT, as well as ascending and descending HL (AHL and DHL) (Figure 7A; Supplementary File S7). Since the proportion of EPT cells was more reduced than the PCT and PST, we analyzed the transcriptome for this cluster and identified 337 upregulated and 22 downregulated genes, with many kidney disease-associated genes among the top downregulated genes (Supplementary Figure S6A; Supplementary File S8). GO analysis revealed that genes involved in metabolic processes, cell-cycle/proliferation, cellular component organization, and negative regulation of cellular process and integrin signaling pathway were upregulated (Supplementary Figure S6B).

We found that certain markers for mature PT cells, including *Ass1*, *Aldob*, and *Slc34a1,* were strongly upregulated in EPT-related cells of *Smarca4*-deficient kidneys. In contrast to control cells, which showed a temporal progression of maturation from EPT to PCT and PST cells based on the upregulation of these genes (Figures 7B, C), *Smarca4*cKO kidneys failed to generate discernible cellular heterogeneity with clearly demarcated populations of EPT and differentiated PT due to elevated expression of these genes in EPT cells (Figures 7B, C). Moreover, the HL/DT-specific genes such as *Wfdc2* (Figure 7C) and *Ly6a* (Supplementary Figure S6A) were also strongly upregulated in *Smarca4*cKO EPT, IM1 and DHL cells, along with many cell-cycle regulators, including *Ki67*, *Cdk1* (Figure 7C), *Cdk2*, *Tab1*, *Pclaf*, *Igfbp5*, *Lmo1*, and *Smc1b* (Supplementary Figure S6C; Supplementary File S8), reflecting higher proliferation rates as seen by anti-PH3 immunostaining (Figure 6B). Upregulation of *Wnt4* was also observed in the mutant IM1 cluster (Supplementary Figure S6C).

In contrast to the upregulation of *Pttg1* in the mutant precursor cells (Figure 6), its expression levels appeared unchanged in these more differentiated tubular cells (Supplementary Figure S6C). However, the expression of ECM genes such as mesenchymal collagens *Col3a1* and *Col1a1* (Figure 7C) as well as EMT markers found in tumorigenic EMT (Latil et al., 2017) or renal fibrosis, including EMT-associated cytoskeleton *Vimentin*, *Fn1* (Figure 7C) and *Sparc* (Supplementary Figure S6C), were significantly increased in *Smarca4*cKO tubular cells. Notably, the hypoxia-inducible TF *Hif1a*, a key regulator for renal fibrosis under various conditions (Liu et al., 2017) was also upregulated in the EPT, PCT and CNT (Figure 7C). Similar to the upregulation of *Col3a1* in the cortical region of *Smarca4*cKO kidneys (Figure 3E), ISH also revealed increased *Fn1* transcripts in the cortical areas and tubular structures (Figure 7D). Co-immunostaining for Vimentin and Ncam (neural cell adhesion molecule) confirmed that *Smarca4*cKO kidneys lacked Ncam-labeled epithelial tubules but had Vimentin^+^ cells in the tubular structures (Figure 7E), indicating the presence of fibrotic cells and induction of genes related to fibrosis. Taken together, these results suggest that although some tubular differentiation genes are expressed in *Smarca4*-deficient kidneys, the mutant tubular cells lose their tubular characteristics due to the induction of cell-cycle regulators and non-tubular ECM/EMT-related genes, leading to cell fibrosis and loss of expression of many nephron tubular-specific genes (Supplementary Figure S6A).

## Discussion

*Wnt4* is one of the earliest genes that mark nephron differentiation, and the *Wnt4* ^*Cre*^ mice have previously been used to delete genes in nephron differentiation. Previous studies have shown that *Wnt4* is expressed in the medullary interstitium (Itaranta et al., 2006; England et al., 2020) and that the Wnt4 signaling is necessary for controlling the fate of SMCs in the kidney, as *Wnt4*-deficient kidneys lack differentiated SMCs (Itaranta et al., 2006). However, how *Wnt4*-expressing cells contribute to renal interstitial development is not fully understood. Here, we used unbiased scRNA-seq analysis to classify the composition and interstitial subtypes from *Wnt4* ^*Cre*^-labeled cells. Our analyses identified a total of 34 clusters: 2 types of immune, 3 types of endothelial and 8 types of stromal cells, SMCs, PCs, UE, 3 types of podocytes, and 15 nephron-related populations. We uncovered previously unknown cell types that had so far not been linked to *Wnt4* ^*Cre*^-labeled cells in the interstitium. Our developmental trajectory analysis revealed that *Wnt4*-expressing CD-associated stromal precursors differentiate into fibroblasts, and then fibroblast progenitors produce some progenitors in the nephrogenic stroma that proliferate and differentiate into pericytes. However, cortical, medullary, ureteric stroma or SMCs appear to be derived from different progenitors. Thus, some transiently amplifying *Wnt4*-expressing progenitors in the interstitium may give rise to precursors to the cortex, medulla, or ureteral stroma, as well as to SMCs. Such precursors or a proportion of them may also express *Foxd1*, which is capable of generating multiple stromal tissues in the kidney. This speculation is consistent with previous observations that *Wnt4*-expressing stromal cells in the medullary region are *Foxd1-*labeled (England et al., 2020) and that *Foxd1*-expressing cortical stroma progenitors are multipotent and are able to give rise to stromal tissues of the interstitium throughout kidney organogenesis (Kobayashi et al., 2014). Nevertheless, these possibilities must be clarified and confirmed using lineage-tracing analysis of *Wnt4* ^*CreER*^.

Cell growth, lineage commitment, differentiation, and genome stability are controlled by the spatiotemporal regulation of genes that are assembled into chromatin. Chromatin remodeling proteins alter local chromatin structure and facilitate recruitment of essential factors required for transcription.

SMARCA4, the central ATPase subunit of numerous chromatin-modifying enzymatic complexes, uses the energy derived from ATP-hydrolysis to disrupt the chromatin architecture of target promoters. It has been implicated in the activation and repression of gene expression through the modulation of chromatin in various tissues and physiological conditions. SMARCA4 can also act as a transcriptional coregulator and interact with tissue- or cell type-specific TFs to regulate gene expression. During development, we found that *Smarca4* is widely expressed in the developing kidney and it interacts with nephron progenitor-specific TFs Eya1 and Six1 to maintain the NPCs (Li et al., 2021). To investigate the role of the SWI/SNF chromatin-remodeling complexes in regulating the differentiation of *Wnt4*-expressing cells, we deleted *Smarca4* using *Wnt4* ^*Cre*^ and compared the sequencing datasets between control and *Smarca4*cKO cells at single-cell resolution. Our scRNA-seq data also revealed *Smarca4* expression in all *Wnt4* ^*Cre*^-labeled cell clusters (Supplementary Figure S6D). The difference between the control and *Smarca4*cKO is that the mutant has increased interstitium but decreased nephron tubule cells, which are unable to maintain tubule-specific characteristics due to elevated proliferative capacity and misexpression of non-tubular genes, including ECM/EMT-related genes. Our findings provide substantial insights into the major role played by *Smarca4* in the cellular proliferation and differentiation of *Wnt4* ^*+*^ cells in the kidney. However, future work is crucial to elucidate the specific impact of *Smarca4* loss on chromatin accessibility in different types of *Wnt4* ^*Cre*^-labeled cells in the kidney. Furthermore, it will be essential to identify the cell type-specific TFs with which Smarca4 interacts to regulate the cell fate and differentiation of *Wnt4* ^*Cre*^-labeled cells.

The BAF complexes are the most frequently mutated chromatin regulatory complexes found in cancers (Hodges et al., 2016). These mutations contribute to various malignancies due to dysregulated cell-cycle control (Kadoch et al., 2013; Shain and Pollack, 2013; Gupta et al., 2020). *SMARCA4* mutations are also associated with Wilms tumors (Rakheja et al., 2014; Treger et al., 2019), a type of renal cancers that accounts for nearly 90% of renal tumors in children and 7% of all childhood cancers (Breslow et al., 1993). Recent studies have shown that *SMARCA4* mutations lead to increased expression of the proto-oncogene *MYC* (Hodges et al., 2018) and the prelicensing protein CDC6 (Gupta et al., 2020). In this study, of particular interest, we found that the deletion of *Smarca4* induced *Pttg1* oncogene upregulation. Pttg1 is involved in cell-cycle progression, transactivation of other oncogenes such as MYC (Pei, 2001), and DNA repair (Vlotides et al., 2007). It is overexpressed in all types of human cancers (Zhang et al., 1999; McCabe et al., 2003; Vlotides et al., 2007), and its increased levels in cancer cells has been shown to cause mis-segregation of chromosomes and facilitate genome instability (Yu et al., 2003). We found that *Pttg1* expression was elevated in the stromal precursors (Figure 4) and the nephron committing and SBB precursors, which are associated with increased proliferation capacity (Figure 6). Although the levels of Pttg1 expression appeared to be unchanged in more differentiated tubular cells (Supplementary Figure S6C), these cells also showed elevated proliferation capacity (Figure 7C; Supplementary Figure S6C). Thus, we speculate that the elevated levels of *Pttg1* in the precursor cells may lead genome instability, resulting in accelerated cell division and induction of ECM/EMT-related genes in the precursor cells. Once the cellular state of these *Wnt4* ^+^ precursors has changed, they will continue to divide rapidly and express ECM/EMT-related genes along with some nephron differentiation genes, a process that does not directly require Pttg1 activity. These precursors fail to transition to a characteristic epithelial tubular state but undergo fibrosis, as evidenced by the lack of expression of many tubular-specific genes and the induction of ECM/EMT markers (Figure 7; Supplementary Figure S6), including the TF Hif1a. During EMT in cells that need to develop a migratory cellular program, transcription must be reactivated to allow for cellular transition. Consistent with this, we observed a global increase in ribosomal transcription in *Smarca4*cKO cells (Supplementary Figure S3A), which is likely necessary for cellular state changes or EMT. The lack of nephron tubular growth and elongation into the medullary region in *Smarca4*cKO may lead to the shortening of the medulla.

In summary, our transcriptome analyses of FACS-sorted *Wnt4* ^*Cre*^-labled tdTomato^+^ cells support previous observations of *Wnt4* expression in medullary stroma cells and its requirement for SMC differentiation. While our results support the previous hypothesis that there may be transiently amplifying *Wnt4*-expressing progenitors in the interstitium that can give rise to multiple precursors, lineage tracing analysis using *Wnt4* ^*CreER*^ at different developmental stages is needed to confirm this possibility. Overall, our findings highlight the critical role of Smarca4 in kidney development and the regulation of *Pttg1* expression, providing new insights into the mechanisms underlying nephron tubule formation and renal disease, including Wilms tumor. The upregulation of the oncogene *Pttg1* in *Smarca4*-deficient kidneys may shed new light on therapeutic strategies for renal cell carcinoma resulting from SWI/SNF complex deficiency.

## Data Availability

scRNA-seq datasets were deposited at the NCBI GEO (https://www.ncbi.nim.nih.gov/geo/) with the accession number GSE200301. All data reported in this paper will be shared by the corresponding author upon request. Any additional information required to reanalyze the data reported in this paper is available upon request.
