# Supplementary material for: Smarca4 deficiency induces Pttg1 oncogene upregulation and hyperproliferation of tubular and interstitial cells during kidney development
Source: Front Cell Dev Biol. 2023 Sep 4;11:1233317. doi: 10.3389/fcell.2023.1233317 (PMC10506413; doi:10.3389/fcell.2023.1233317)
Supplement: Supplementary file 4 [file DataSheet1.PDF]

Supplemental Figure 1. Deletion of *Smarca4* using *Wnt4*$^{Cre/+}$ leads to smaller kidneys. (A) Anti-Smarca4 immunostaining. Arrows indicate nephron structures and depletion of Smarca4 in the mutant. Note that Smarca4 expression in some RVs were not completely depleted. (B) Kidneys of *Smarca4*$^{fl/fl}$, *Wnt4*$^{Cre/+}$ and *Smarca4*$^{cKO/cKO}$ littermates at E18.5. Abb.: CD, collecting duct; K, kidney; SSB, S-shaped body.

Supplemental Figure 2. scRNA-seq analysis of *Wnt4*-labeled cells and trajectory analysis of endothelial cells. (A) Flow cytometry plots showing gates and regions distinguishing tdTomato-negative (from R26-tdTomato kidneys) and tdTomato-positive cells. (B) UMAP plots showing 24 clusters (11 major groups) of *Wnt4*-labeled cells of control and *Smarca4*$^{cKO/cKO}$. (C) Percentages of assigned cell groups. Colored fonts indicate representative clusters of increased (red) or decreased (blue) cell numbers in the mutant. (D) UMAP plots showing high levels of *Six2* expression in nephron progenitors, *Pcp4* in podocyte precursors (CS11), *Nphs2* in mature podocytes (CS15), *Nphs1* in mature (CS15) and immature (CS22) podocytes. (E) UMAP plots showing high levels of high levels of *Rgcc* in endothelial cluster 2 and 7, *Cdk1* in endothelial cluster 7, *Gja4* in endothelial cluster 14, and *Tyrobp* in immune cells. (F) UMAP representations of scRNA-seq endothelial cell differentiation trajectory from the proliferative endothelial cluster 7. Cells are colored by pseudotime.

Supplemental Figure 3. Deletion of *Smarca4* in *Wnt4*-expressing cells leads to global upregulation of genes involved in cell-cycle/cell proliferation and trajectory analysis of nephron tubule differentiation. (A) Violin plots showing global upregulation of *Gm10260*, *Tpm3-rs7* and *Hist1h2ap* and upregulation of *Col3a1* in some clusters in *Smarca4*cKO cells. (B) Cellular trajectory of nephron tubule differentiation. Cells are colored by pseudotime. The circles with

numbers denote special points within the graph. Each leaf, denoted by light gray circles, corresponds to a different outcome (i.e. cell fate) of the trajectory. Black circles indicate branch nodes where cells can travel to one of several outcomes. The numbers within the circles are provided for reference purposes only.

Supplemental Figure 4. Clustering of *Wnt4*-derived stromal cells and SMCs in control and *Smarca4*cKO kidneys. (A) Heatmap of *Wnt4*-labeled stromal subtypes and SMCs by top 4 marker genes in control and *Smarca4*cKO. (B) UMAP plots showing *Tbx18, Itm2a, Mki67* and *Acta2* expression in ureteric stroma. Arrowheads point to the *Itm2a*$^{+}$ stroma in the mutant. (C) Cellular trajectory of stromal cells using fibroblasts as root cells. Cells are colored by pseudotime. The circles with numbers denote special points within the graph. Each leaf, denoted by light gray circles, corresponds to a different outcome (i.e. cell fate) of the trajectory. Black circles indicate branch nodes where cells can travel to one of several outcomes. The numbers within the circles are provided for reference purposes only. Note the lack of black circles in the control using nephrogenic stroma as the root.

Supplemental Figure 5. Deletion of *Smarca4* disrupts early patterning of SSBs. **(A)** Whole-mount *in situ* hybridization reveals reduced nephron tubular primordial structures in *Smarca4*cKO kidneys. Images of E15.0 kidneys *in situ* hybridized with *Lhx1*, *Dll1*, *Pou3f3*, *Irx1* and *Irx2* riboprobe respectively in *Wnt4*$^{Cre/+}$ and *Smarca4*$^{cKO/cKO}$ littermate embryos. Clustering of *Wnt4*-lineage-derived uncommitted and committed nephron progenitors and SSB precursors in control and *Smarca4*cKO. (B) Violin plots showing *Notch1*, *Notch2, Hnf1b* and *Mafb* in distinct types of nephron precursors. Boxed area showing decreased *Nocth1* expression

in the intermediate region (for PT fate) of *Smarca4*cKO SSB. (C) UMAP plots showing high levels of *Six2* in uncommitted, *Wnt4* in committing nephron progenitors, and increased expression of *Pttg1* and *Top2a* in the committing NP, proximal and intermediate regions of SSB and NP-stroma (arrows).

Supplemental Figure 6. Clustering of *Wnt4*-lineage-derived nephron tubule cells and increased cell proliferation in *Smarca4*cKO and *Smarca4* expression in all cell clusters. (A) Violin plots showing altered expression of renal disease-associated genes in distinct tubular regions in *Smarca4*cKO. (B) GO analysis of top DEGs in the EPT cluster showing enriched GO terms related to biological process and pathways. (C) Violin plots showing increased expression of cell proliferation markers *Cdk2, Tab1, Pclaf,* and *Igfbp5,* upregulation of *Wnt4* and cell-cycle regulators *Lmo1* and *Smc1b* in IM2, and mesenchymal ECM gene *Sparc* in EPT, PCT, CNT and DCT in *Smarca4*cKO. However, *Pttg1* expression did not appear to be increased in these more differentiated nephron tubular cells. (D) UMAP plot showing *Smarca4* expression in all $\mathit{Wnt4}^{\mathit{Cre}}$-labeled cell clusters in control kidneys.

Supplemental File 1. Full lists of cluster markers for $\mathit{Wnt4}^{\mathrm{Cre}}$-labeled cells in control kidneys.

Supplemental File 2. Full lists of cluster DEGs between S*marca4*cKO and control.

Supplemental File 3. Full lists of cluster markers for *Wnt4*-derived stromal and smooth-muscle cells in control and S*marca4*cKO kidneys.

Supplemental File 4. Full lists of cluster DEGs for *Wnt4*-derived stromal and smooth-muscle cells between S*marca4*cKO and control.

Supplemental File 5. Full lists of cluster markers for *Wnt4*-derived nephron progenitors and committing and SSB precursors in control and S*marca4*cKO kidneys.

Supplemental File 6. Full lists of cluster DEGs for *Wnt4*-derived nephron progenitors and committing and SSB precursors between S*marca4*cKO and control.

Supplemental File 7. Full lists of cluster markers for *Wnt4*-derived nephron tubule cells in control and S*marca4*cKO kidneys.

Supplemental File 8. Full lists of cluster DEGs for *Wnt4*-derived nephron tubule cells between S*marca4*cKO and control.

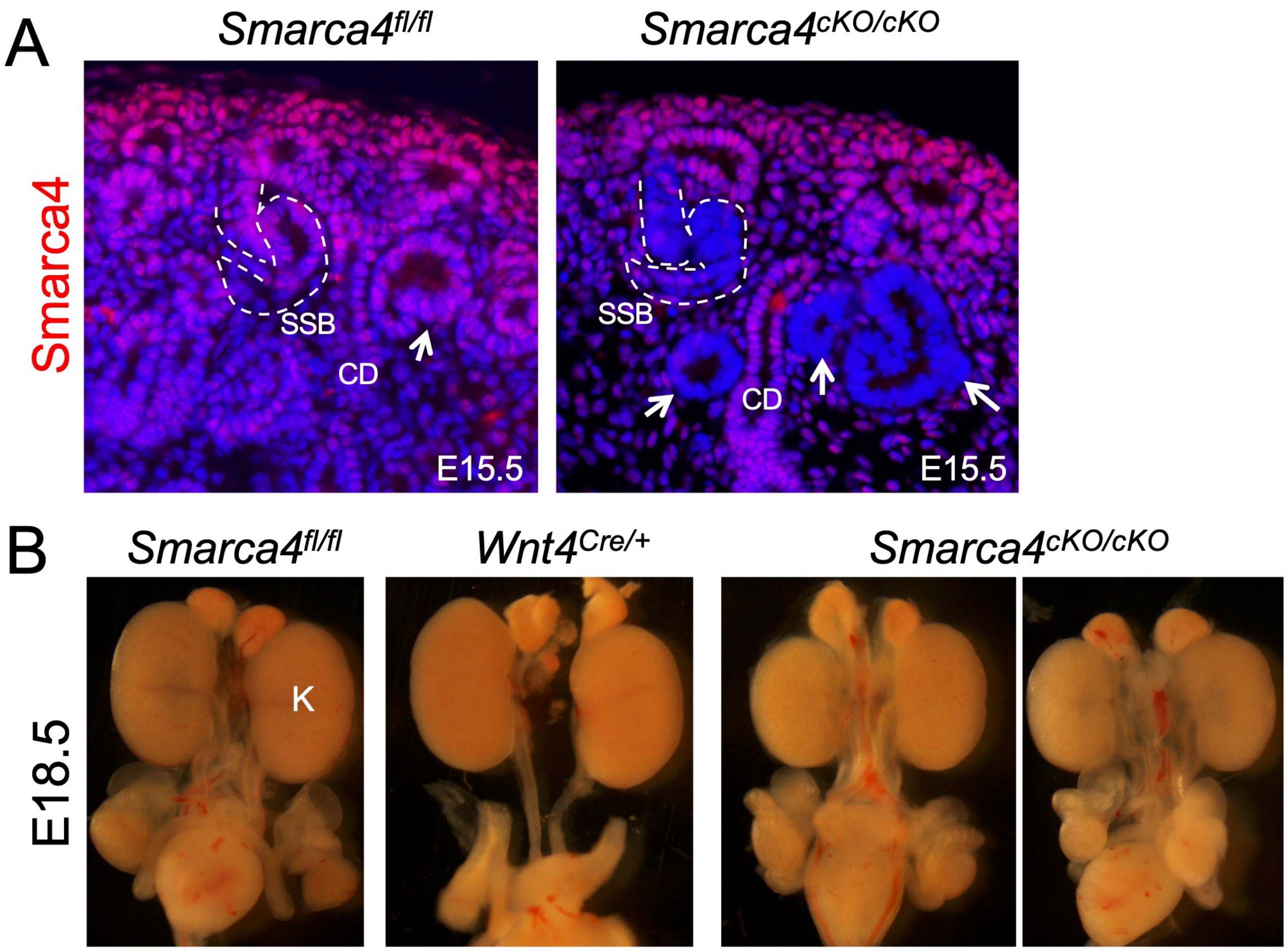
A
Smarca4fl/fl
Smarca4cKO/cKO
Smarca4
SSB
CD
E15.5
SSB
CD
E15.5
B
Smarca4fl/fl
Wnt4Cre/+
Smarca4cKO/cKO
E18.5
K

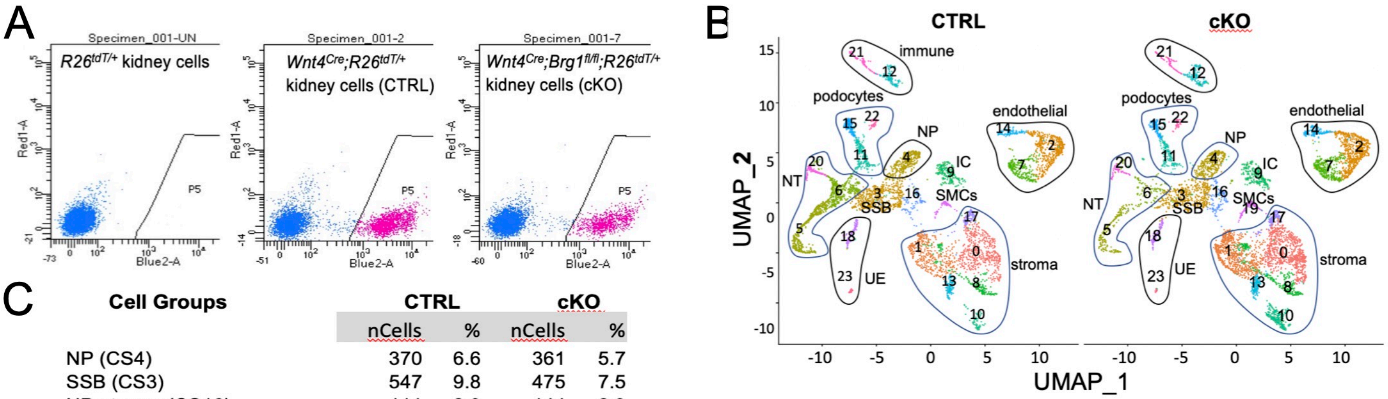

C

Cell Groups	CTRL nCells	CTRL %	cKO nCells	cKO %
NP (CS4)	370	6.6	361	5.7
SSB (CS3)	547	9.8	475	7.5
NP-stroma (CS16)	111	2.0	144	2.3
Nephron tubule (CS5,6,20)	1071	8.2	390	2.4
Podocytes (CS11,15,22)	465	8.3	314	5.0
Intercalated cell (IC, CS9)	232	4.2	280	4.4
Immune cell (CS12,21)	254	4.6	253	4.0
SMCs (CS19)	69	1.2	138	2.2
Endothelial (CS2,7,14)	847	15.2	1170	18.6
Stroma (CS0,1,8,10,13,17)	1435	25.7	2374	37.7
UE (CS18,23)	178	3.19	132	2.1
Total number of cells	5579	100.0	6299	100.0

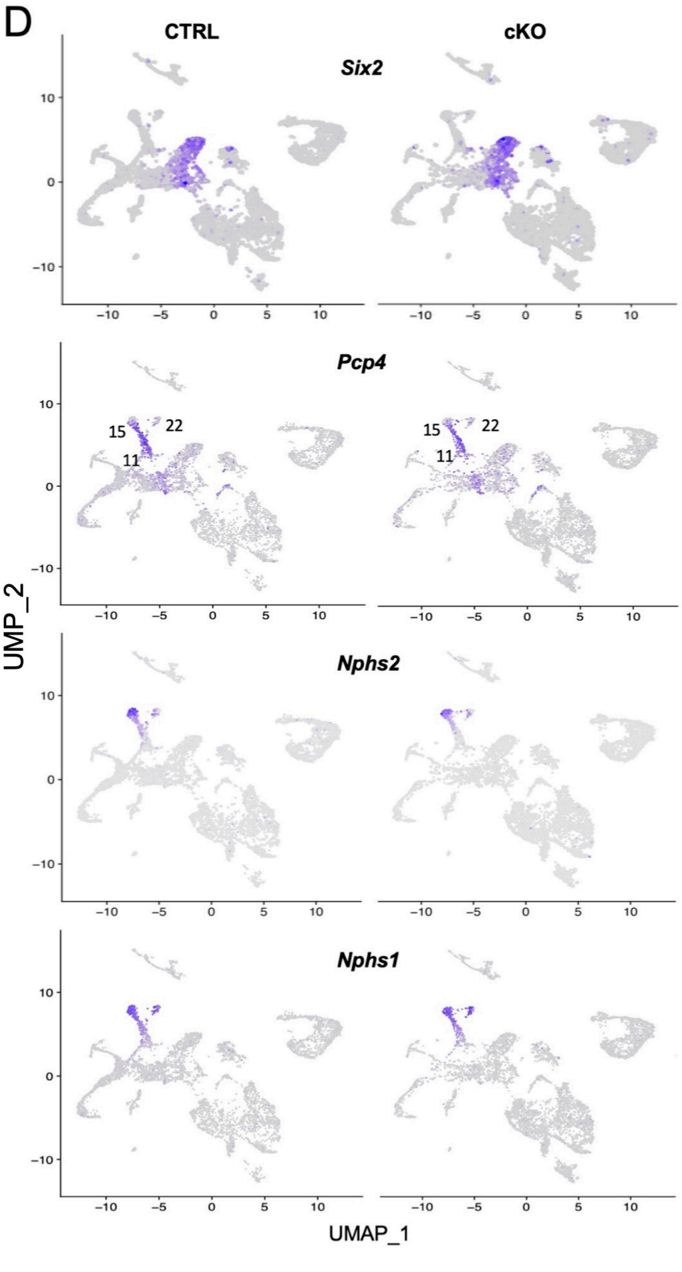

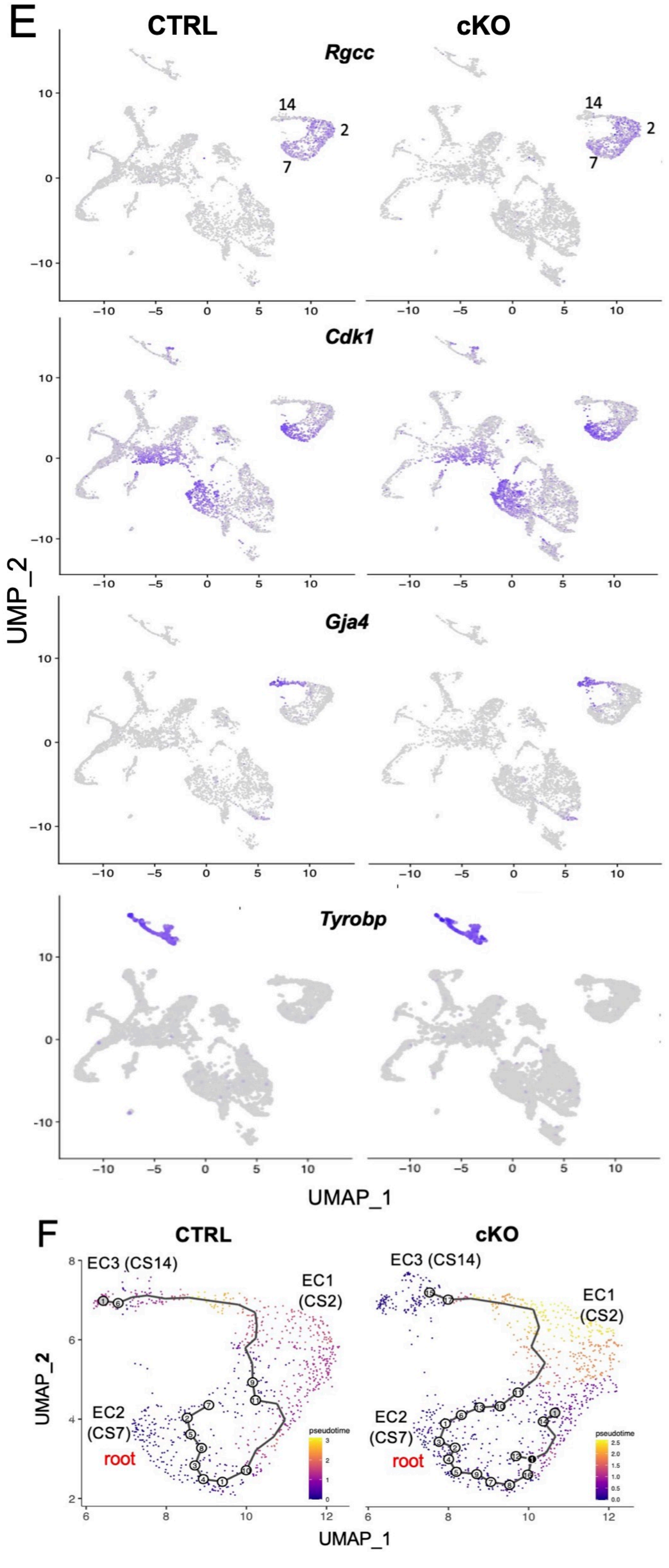

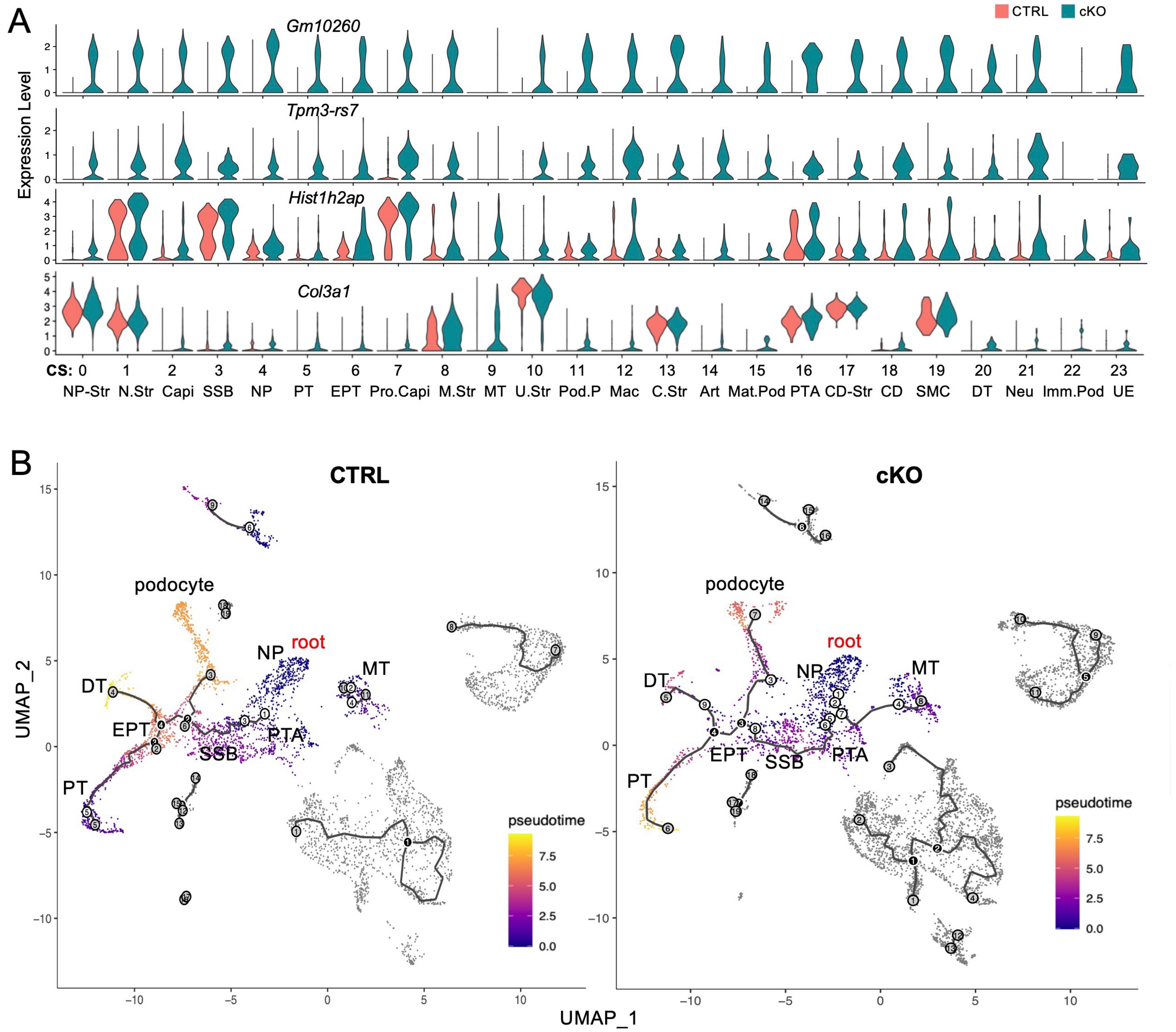
A
CTRL
cKO
Gm10260
Tpm3-rs7
Hist1h2ap
Col3a1
Expression Level
CS: 0 1 2 3 4 5 6 7 8 9 10 11 12 13 14 15 16 17 18 19 20 21 22 23
NP-Str N.Str Capi SSB NP PT EPT Pro.Capi M.Str MT U.Str Pod.P Mac C.Str Art Mat.Pod PTA CD-Str CD SMC DT Neu Imm.Pod UE
B
CTRL
cKO
podocyte
root
NP
MT
DT
EPT
PTA
SSB
PT
pseudotime
UMAP_2
UMAP_1

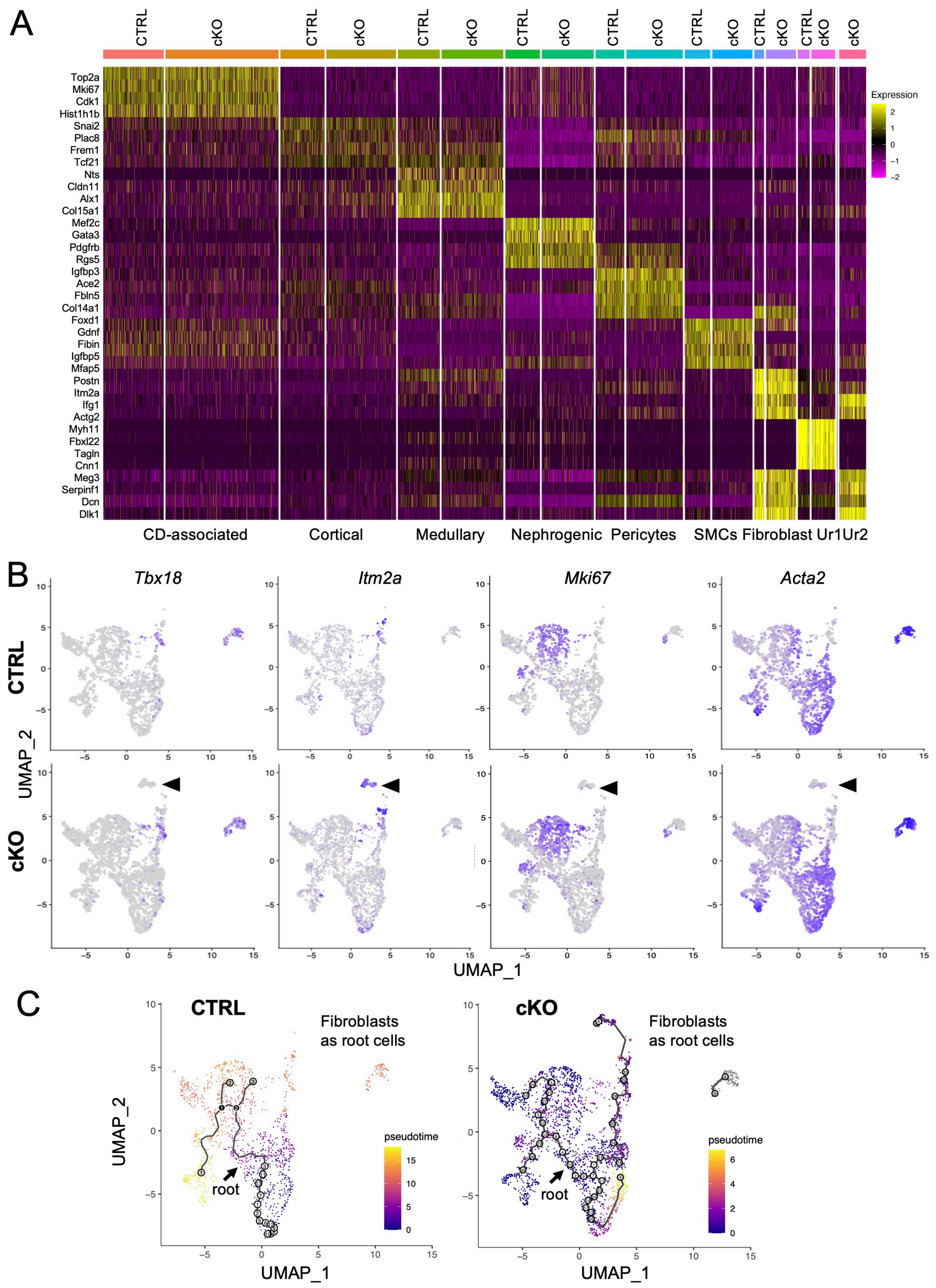
A
CTRL
cKO
Top2a
Mki67
Cdk1
Hist1h1b
Snai2
Plac8
Frem1
Tcf21
Nts
Cldn11
Alx1
Col15a1
Mef2c
Gata3
Pdgfrb
Rgs5
Igfbp3
Ace2
Fbln5
Col14a1
Foxd1
Gdnf
Fibin
Igfbp5
Mfap5
Postn
Itm2a
Ifg1
Actg2
Myh11
Fbxl22
Tagln
Cnn1
Meg3
Serpinf1
Dcn
Dlk1
Expression
CD-associated
Cortical
Medullary
Nephrogenic
Pericytes
SMCs
Fibroblast
Ur1
Ur2
B
Tbx18
Itm2a
Mki67
Acta2
CTRL
cKO
UMAP_2
UMAP_1
C
CTRL
cKO
Fibroblasts as root cells
root
pseudotime
UMAP_2
UMAP_1

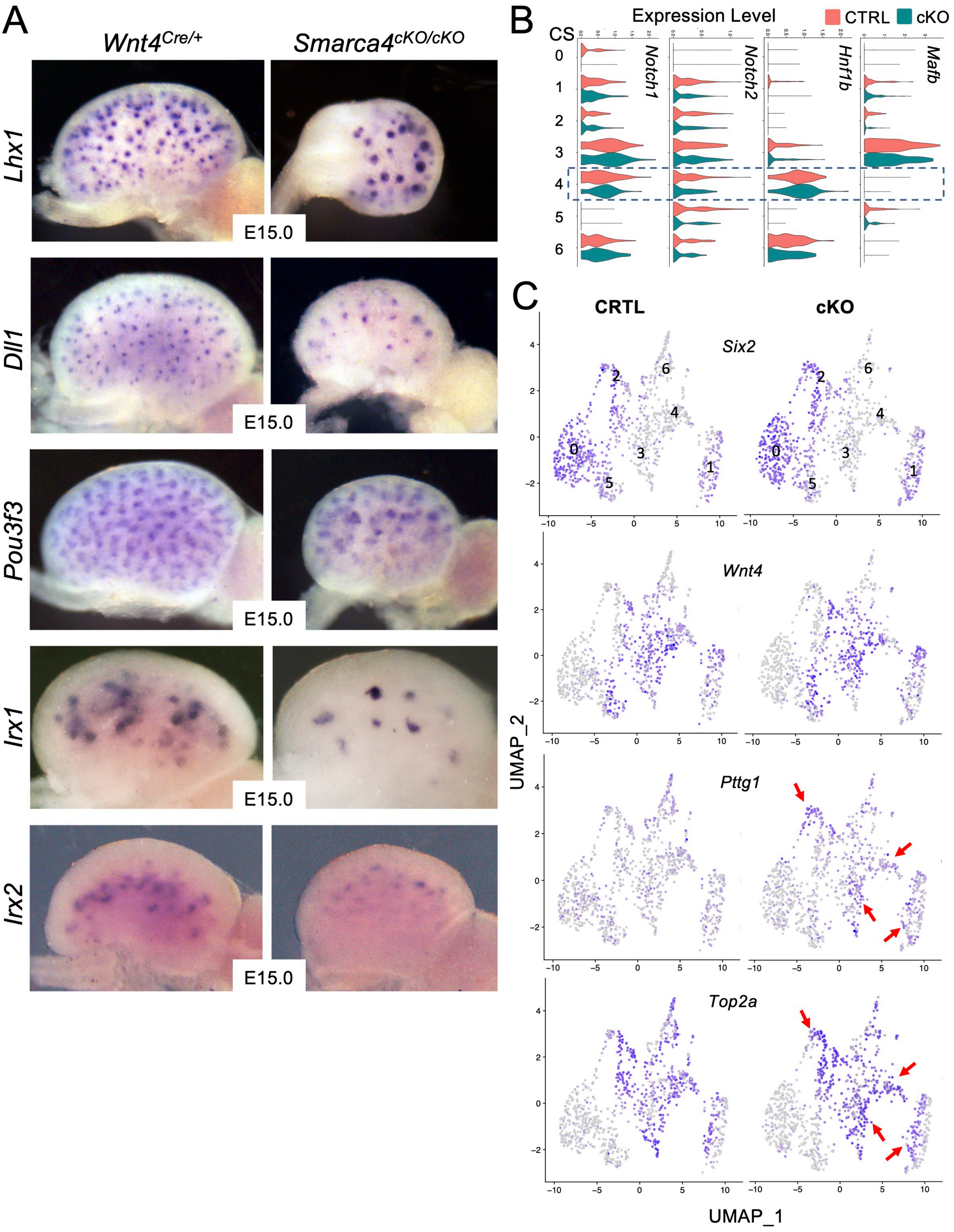
A
Wnt4$^{Cre/+}$
Smarca4$^{cKO/cKO}$
Lhx1
Dll1
Pou3f3
Irx1
Irx2
E15.0
B
Expression Level
CTRL
cKO
CS
Notch1
Notch2
Hnf1b
Mafb
C
CRTL
cKO
Six2
Wnt4
Pttg1
Top2a
UMAP_2
UMAP_1

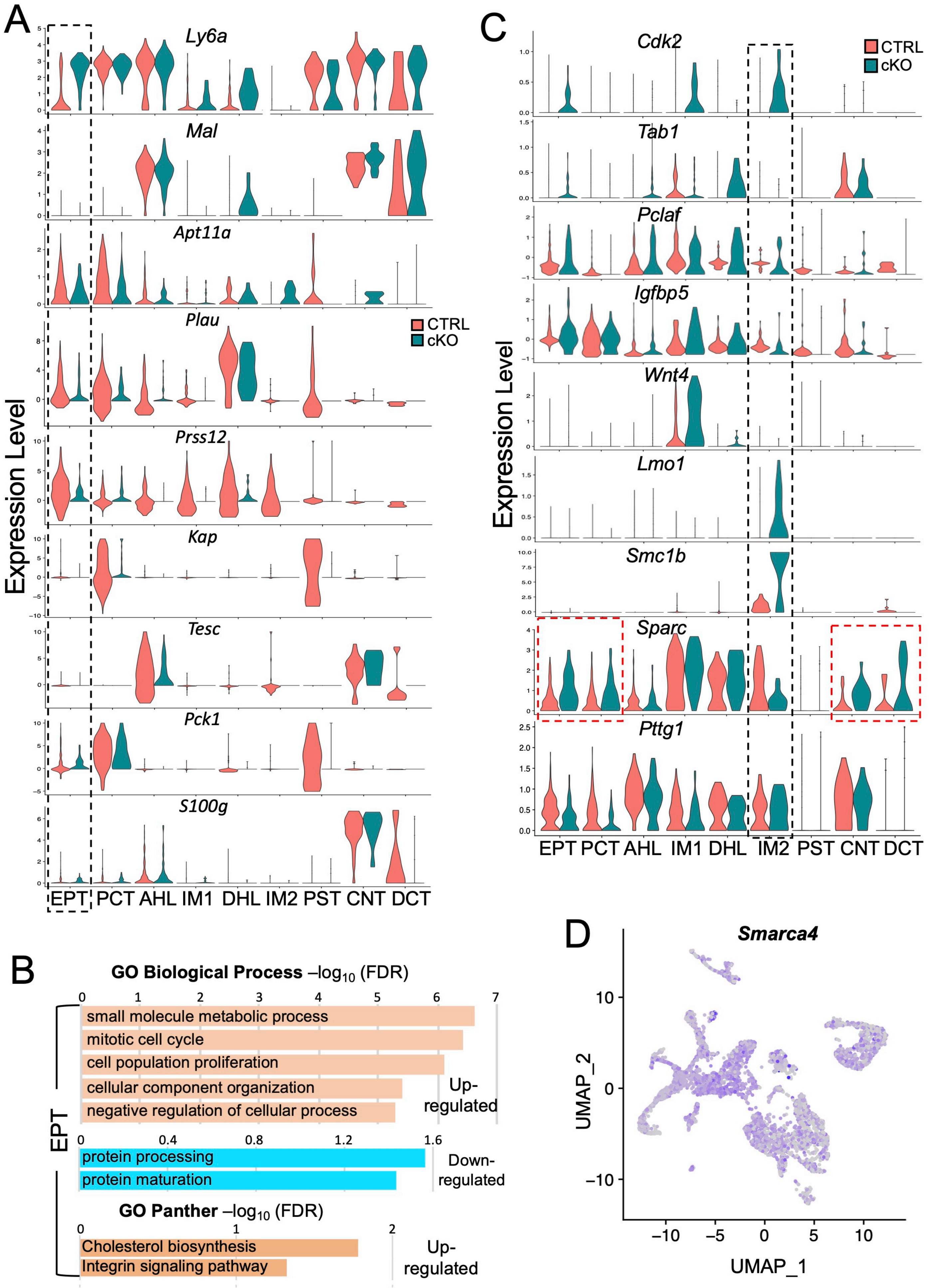

A
Ly6a
Mal
Apt11a
Plau
Prss12
Kap
Tesc
Pck1
S100g
CTRL
cKO
Expression Level
EPT PCT AHL IM1 DHL IM2 PST CNT DCT
C
Cdk2
Tab1
Pclaf
Igfbp5
Wnt4
Lmo1
Smc1b
Sparc
Pttg1
CTRL
cKO
Expression Level
EPT PCT AHL IM1 DHL IM2 PST CNT DCT
B
GO Biological Process –log10 (FDR)
small molecule metabolic process
mitotic cell cycle
cell population proliferation
cellular component organization
negative regulation of cellular process
Up-regulated
EPT
protein processing
protein maturation
Down-regulated
GO Panther –log10 (FDR)
Cholesterol biosynthesis
Integrin signaling pathway
Up-regulated
D
Smarca4
UMAP_2
UMAP_1
